# Reinvestigation of Aminoacyl-TRNA Synthetase Core Complex by Affinity Purification-Mass Spectrometry Reveals TARSL2 as a Potential Member of the Complex

**DOI:** 10.1371/journal.pone.0081734

**Published:** 2013-12-02

**Authors:** Kyutae Kim, Seong-Jun Park, Seungjin Na, Jun Seok Kim, Hyungwon Choi, Yoon Ki Kim, Eunok Paek, Cheolju Lee

**Affiliations:** 1 Biomedical Research Institute, Korea Institute of Science and Technology, Seongbuk-gu, Seoul, Korea; 2 School of Life Sciences and Biotechnology, Korea University, Seongbuk-gu, Seoul, Korea; 3 Division of Computer Science and Engineering, Hanyang University, Seongdong-gu, Seoul, Korea; 4 Saw Swee Hock School of Public Health, National University of Singapore, Singapore, Singapore; 5 Department of Biological Chemistry, University of Science and Technology, Daejeon, Korea; The Research Institute for Children, United States of America

## Abstract

Twenty different aminoacyl-tRNA synthetases (ARSs) link each amino acid to their cognate tRNAs. Individual ARSs are also associated with various non-canonical activities involved in neuronal diseases, cancer and autoimmune diseases. Among them, eight ARSs (D, EP, I, K, L, M, Q and RARS), together with three ARS-interacting multifunctional proteins (AIMPs), are currently known to assemble the multi-synthetase complex (MSC). However, the cellular function and global topology of MSC remain unclear. In order to understand the complex interaction within MSC, we conducted affinity purification-mass spectrometry (AP-MS) using each of AIMP1, AIMP2 and KARS as a bait protein. Mass spectrometric data were funneled into SAINT software to distinguish true interactions from background contaminants. A total of 40, 134, 101 proteins in each bait scored over 0.9 of SAINT probability in HEK 293T cells. Complex-forming ARSs, such as DARS, EPRS, IARS, Kars, LARS, MARS, QARS and RARS, were constantly found to interact with each bait. Variants such as, AIMP2-DX2 and AIMP1 isoform 2 were found with specific peptides in KARS precipitates. Relative enrichment analysis of the mass spectrometric data demonstrated that TARSL2 (threonyl-tRNA synthetase like-2) was highly enriched with the ARS-core complex. The interaction was further confirmed by coimmunoprecipitation of TARSL2 with other ARS core-complex components. We suggest TARSL2 as a new component of ARS core-complex.

## Introduction

Aminoacylation reaction catalyzed by aminoacyl-tRNA synthetases (ARSs) is the first step in protein production. Amino acids are covalently attached to its cognate tRNA. Among 20 ARSs, eight different ARSs (aspartyl-tRNA synthetase (DARS), bifunctional glutamyl-prolyl-tRNA synthetase (EPRS), Isoleucyl-tRNA synthetase (IARS), lysyl-tRNA synthetase (KARS), leucyl-tRNA synthetase (LARS), methionyl-tRNA synthetase (MARS), glutaminyl-tRNA synthetase (QARS) and arginyl-tRNA synthetase (RARS)) are known to form a multisynthetase complex (MSC) together with three ARS-interacting multifunctional proteins (AIMPs)[1]. Although the cellular function of the MSC remains unclear, a number of possible functions have been suggested. First, MSC may increase the efficiency of protein biosynthesis by providing a channel for tRNAs [2]. Second, the complex could act as a molecular reservoir to control non-canonical activities of ARSs [3]. In addition, they have been proposed to help stabilize translation components and promote tRNA transportation to the cytoplasm [4].

Accumulating evidence suggests that various functions of complex-forming ARSs, members of MSC, as well as non-complex forming ARSs are systematic and are controlled through sophisticated mechanisms in response to various cellular stimuli [5]. For example, AIMP1 mainly plays a scaffolding role in assembly of MSC. But, it is also secreted outside of the cell and works as a cytokine on various target cells such as endothelial cells, dendritic cells, fibroblasts, etc [6-8]. AIMP2 also participates in additional activities apart from the MSC, such as suppression of cell proliferation and apoptosis induction by activating p53 or mediating TNF-alpha signal [9-11]. KARS shows the most diverse activities so far. First, secretion of KARS induced by TNF-alpha (tumor necrosis factor-alpha) activates macrophages to enhance TNF-alpha production and it helps growth of cancer cells [12]. Under specific stimuli, KARS is serine-phosphorylated in a MAPK (mitogen-activated protein kinase)-dependent manner, dissociates from MSC and translocates from the cytoplasm to the nucleus. The released KARS produces higher level of Ap_4_A (diadenosine tetraphosphate), with profound cellular effects via binding to Ap_4_A-binding proteins. One such effect is removal of repressor Hint-1 (histidine triad nucleotide binding protein 1) from MITF (microphthalmia-associated transcription factor), enabling it to transcribe its target genes. Consequently, KARS has a signal transduction role besides its other well-defined roles in immunologically activated cells [13].

Many approaches have been attempted to get insights into the molecular networks of protein interaction, such as yeast two-hybrid analysis, pull-down assay and systematic depletion studies. Recently, affinity purification coupled to mass spectrometry (AP-MS) has become the method of choice for protein complex characterization with the improved performance in tandem mass spectrometry (MS/MS) technology and affinity purification strategies [14]. One benefit of AP-MS is that it can identify not only binary interactions, but also entire protein complexes. Another advantage of AP-MS is to identify post-translational modifications (PTMs), such as phosphorylation and acetylation. Therefore, it can provide information about signaling pathways (reviewed in ref [15].). Protein affinity tags are widely used for protein purification, in particular, from complex mixtures such as lysed cells. Among various affinity purification methods using GST (glutathione S-transferase), MBP (maltose binging protein), FLAG, SBP (streptavidin binding peptide), and His tags, SBP tag offers superior purity and yield in various expression systems. Purification using SBP-tag is suitable for high-throughput protein expression and purification procedures [16]. After AP-MS analysis, identification of true interactions from background contaminants is important for biological research. In order to distinguish the true interactions, several groups have developed such approaches as Normalized Spectral Abundance Factor (NSAF) and Comparative proteomic analysis software suite (CompPASS), in which resultant scores are empirical transformations of spectral counts without a probability model [17,18]. In contrast, a recently developed probability-based algorithm, Significance Analysis of INTeractome (SAINT) assigns confidence scores to protein-protein interaction by constructing separate distributions for true and false interactions using spectral counts [19].

Taking advantage of AP-MS and SAINT algorithm, we investigated the protein-protein interaction of ARS-MSC in order to extend the current knowledge by discovering novel interacting proteins using the SBP tag. We used each of AIMP1, AIMP2 and KARS as a bait for the AP-MS experiments. We also sought to predict the function of MSC systematically. From this study, we expect a comprehensive understanding of ARS core interactome, which will be great resource to understand its biological functions.

## Materials and Methods

### AIMP1, AIMP2 and KARS Transfection

AIMP1 gene in pET28a vector, AIMP2 in pET28a and KARS in pEG202 were provided by Medical Bioconvergence Research Center (Gyeonggi, Korea). AIMP1 and KARS were cloned into a vector pIRES2-EGFP-SBP, engineered to express fusion proteins with N-terminal S, FLAG and SBP tag. AIMP2 gene was amplified by PCR in order to insert SalΙ/BamHΙ sites and cloned into the pIRES2-EGFP-SBP. The resulting vectors were transiently transfected into HEK 293T and HCT-8 cell lines purchased from American Type Culture Collection (ATCC). As a negative control, a mock vector having only the S/FLAG/SBP tag was also transfected into the cell lines. After incubating 30 h, cells were harvested at a confluence of 90~100% and lysed by NETN buffer containing 20 mM Tris-HCl, pH 7.4, 1 mM EDTA, 150 mM NaCl, 0.5% Nonidet P-40, Protease Inhibitor Cocktail (Roche Diagnostics) and Phosphatase Inhibitor Cocktail (Roche Diagnostics). Cell debris was pelleted by centrifugation (12,000 rpm, 10 min, 4 °C) and the supernatant was collected. Then, protein amount was measured by Bradford assay and the protein expression was confirmed by immunoblotting.

### Affinity Purification

For Streptavidin pull down (SA pull down), 60 μl of streptavidin agarose beads (Thermo Scientific) in Phosphate buffered-Saline (PBS) was activated and equilibrated by 600 μl of NETN buffer twice. Subsequently, 2 mg of protein was added to the agarose bead and mixed by a rotator (10 rpm, 2 h, 4 °C). After incubation, the beads were washed three times with NETN buffer. Finally, bound proteins were incubated on the top of a 0.22 μm PVDF filter (Millipore, Billerica, MA) for 2 min on ice with 30 μl of biotin solution (approx. 0.82 mM Biotin in NETN buffer) and were eluted by centrifugation (2,000 rpm, 2 min, 4 °C). The elution step was repeated twice. One-tenth of elution was visualized by SDS-PAGE and silver staining. The remaining 90% of eluted proteins was shortly run on the SDS-PAGE and the gel was stained with Coommassie Brilliant Blue solution containing ethanol instead of methanol [20].

### In-Gel Digestion

Each lane of gels was sliced into three equal pieces and destained with ethanol [20]. Proteins in gel slices was reduced with 10 mM dithiothreitol at 56 °C for 1 h and alkylated with 55 mM iodoacetamide in the dark for 1 h at 25 °C. After dehydrating the gel pieces, rehydration was performed by adding 30 μl of trypsin (0.0125 μg/μl) for 30 min at 4 °C. The gel pieces were cautiously washed with 25 mM ammonium bicarbonate twice and incubated with 50 mM ammonium bicarbonate at 37 °C overnight. Supernatants were collected, and gels were extracted with 80 μl of 50% acetonitrile and 25 mM ammonium bicarbonate, then with 80 μl of 50% acetonitrile and 0.1% trifluoroacetic acid (TFA), and finally with 80 μl of 70% acetonitrile and 0.1% TFA. All extracts were combined and dried in vacuo. The samples were desalted with a C18 spin column (Thermo Scientific, #NC169595).

### LC-MS/MS

NanoLC-MS/MS experiments were performed on a Multi-Dimensional Liquid Chromatography system (Eksigent) connected to an LTQ XL-Orbitrap mass spectrometer (Thermo Scientific) through a nanospray ion source. The peptide samples were reconstituted in 5 μl of 0.4% acetic acid and 2 μl of each sample was loaded onto a reversed-phase analytical column (15 cm x 75 μm) packed with MAGIC 18aq resin (5 μm, 200Å; Michrom Bioresources). The column was equilibrated with 95% buffer A (0.1% formic acid in H_2_O) + 5 % buffer B (0.1% formic acid in acetonitrile) prior to use. Peptides were eluted at a flow rate of 300 nL/min with a linear gradient of 10 to 40% buffer B over 40 min. The spray voltage was set to 1.9 kV, and the temperature of the heated capillary was set to 250 °C. The LTQ-XL Orbitrap instrument was operated in the data dependent mode. Full-scan MS spectra (m/z 300~2,000) were acquired in the Orbitrap with 1 microscan and a resolution of 100,000 allowing the preview mode at 7,500 resolution for precursor selection and charge-state determination. MS/MS spectra of the five highest-intensity precursor ions were acquired in the ion-trap. Typical mass spectrometric conditions were as follow: ion selection threshold, 500; isolation width, 2 Da; normalized collision energy, 35%; activation Q, 0.25; activation time, 30 ms; dynamic exclusion duration, 40 s. Precursors with unmatched charge states were discarded during data-dependent acquisition. Data were acquired using Xcalibur software v2.0.7.

### Database Search

Raw data files of MS/MS spectra (.raw) were converted to MASCOT generic files (.mgf) using msconvert module in Trans-Proteomic Pipeline (TPP, version 4.5). The mgf peak lists were searched by MASCOT search engine (v.2.3.01; Matrix Science) against the International Protein Index human database (IPI human, version 3.87, European Bioinformatics Institute, http//www.ebi.ac.uk/IPI). The mass tolerance of precursor ions and fragment ions was 15 ppm and 0.5 Da, respectively. One missed cleavage was allowed and peptides with at least 7 amino acids were retained. Variable modification of methionine oxidation and a fixed modification of carbamidomethylation on cysteine residue were allowed. MASCOT MS/MS ion search results (.dat) were converted to XML file using Mascot2XML module of TPP and PeptideProphet/ProteinProphet were performed on the pepXML files for validation of identified peptides and protein grouping. ProteinProphet probability 0.9 corresponded to 0.8% false discovery rate (FDR). In parallel, MASCOT Error Tolerant search was conducted to extend the search space to various modifications. Resulting peptides were filtered with a significance threshold of p < 0.05. The cutoff ion score for peptide identification was 26 or 27 depending on the dataset. These scores corresponded to p-value 0.05. Then protein family was filtered with the followings: significance threshold p < 0.05; maximum number of families, auto; ion score of expect cut-off, 26 or 27. Results were exported into csv files and further cut off by the acquired number of spectra more than or equal to two. The NSAF values were extracted using Abacus [21] with the PeptideProphet probability threshold 0.5 and ProteinProphet probability threshold 0.9. 

MODa (v. 1.02) [22], a blind modification search tool, searches were conducted to cross-check the peptide modifications discovered by MASCOT. MODa was executed with its mass tolerance of precursor ions set to be flexible so that it can compensate for isotope errors and automatically correct such errors, while 0.5 Da was used for the mass tolerance of fragment ions. Peptides were assumed to be possibly modified up to ±200 Da, the search was conducted with no enzyme specificity, and any number of modifications per peptide was allowed during the search. Finally, the peptide identifications were obtained at FDR 1% using a target-decoy strategy, where randomly shuffled sequences were used as a decoy in the search.

### Bioinformatic Analyses

SAINT assigns a confidence score to each interaction, computed as the probability of true interactions given the spectral count data from real bait and control. Using its most recent implementation SAINTexpress (http://saint-apms.sourceforge.net/), we analyzed the purification data for AIMP1, AIMP2 and KARS with appropriate negative controls. SAINT probabilities can be used to estimate FDR. Threshold 0.9 was approximately equivalent to an estimated FDR of 2% [19]. Therefore, the prey proteins assigned SAINT score 0.9 or above were searched against UniProt and EBI QuickGO databases via Software Tool for Rapid Annotation of Proteins (STRAP, version 1.1.0.0) [23]. GO analysis results were exported from STRAP and visualized in Excel. To get insight into the known and predicted protein-protein associations, datasets were applied to STRING (Search Tool for the Retrieval of Interacting Genes/Protein, version 9.05) and mapped with high confidence (>0.7).

### Coimmunoprecipitation and Immunoblotting

For immunoprecipitation and immunoblotting, cells were harvested, washed with chilled PBS, and lysed in NETN buffer. Cell lysate was centrifuged and the supernatant was incubated with primary antibodies for 2 h at 4 °C and further incubated for additional 12 h after adding protein A/G PLUS-Agarose (Santa Cruz Biotechnology). Then, the beads were collected by centrifugation at 2,500 rpm for 3min, washed three times with NETN buffer and resuspended with 2× SDS-PAGE sample buffer. After boiling for 5 min, proteins were separated by SDS-PAGE and then transferred to a PVDF membrane (Bio-Rad) using a Bio-Rad Trans-blot Cell system (Bio-Rad). Electrophoretic transfer to the PVDF membrane was performed at 300 mA for 1 hr. Non-specific binding sites on the membrane were blocked by incubation with 5% skim milk for 1 h at room temperature (RT). Then, the membrane was incubated with primary antibodies at 4 °C overnight. The membrane was washed three times with TBS-T buffer and then incubated with secondary antibody for 1 hour at RT. Immunoreactive proteins were detected using ECL plus (GE Healthcare). The primary antibodies used in the current study were directed against the following proteins: FLAG (F3165, Sigma-Aldrich); normal mouse IgG (sc-2025), normal rabbit IgG (sc-2027) (Santa Cruz Biothecnology); TARSL2 (threonyl-tRNA synthetase like-2; ab93186), TARS (ab58240), EPRS (ab31531), AIMP1 (ab96506), AIMP2 (ab101840), KARS (ab129080), ISG15 (ab92345) (Abcam). 

## Results

### Affinity Purification of S/FLAG/SBP Tagged AIMP1, AIMP2 and KARS

**Figure 1:**
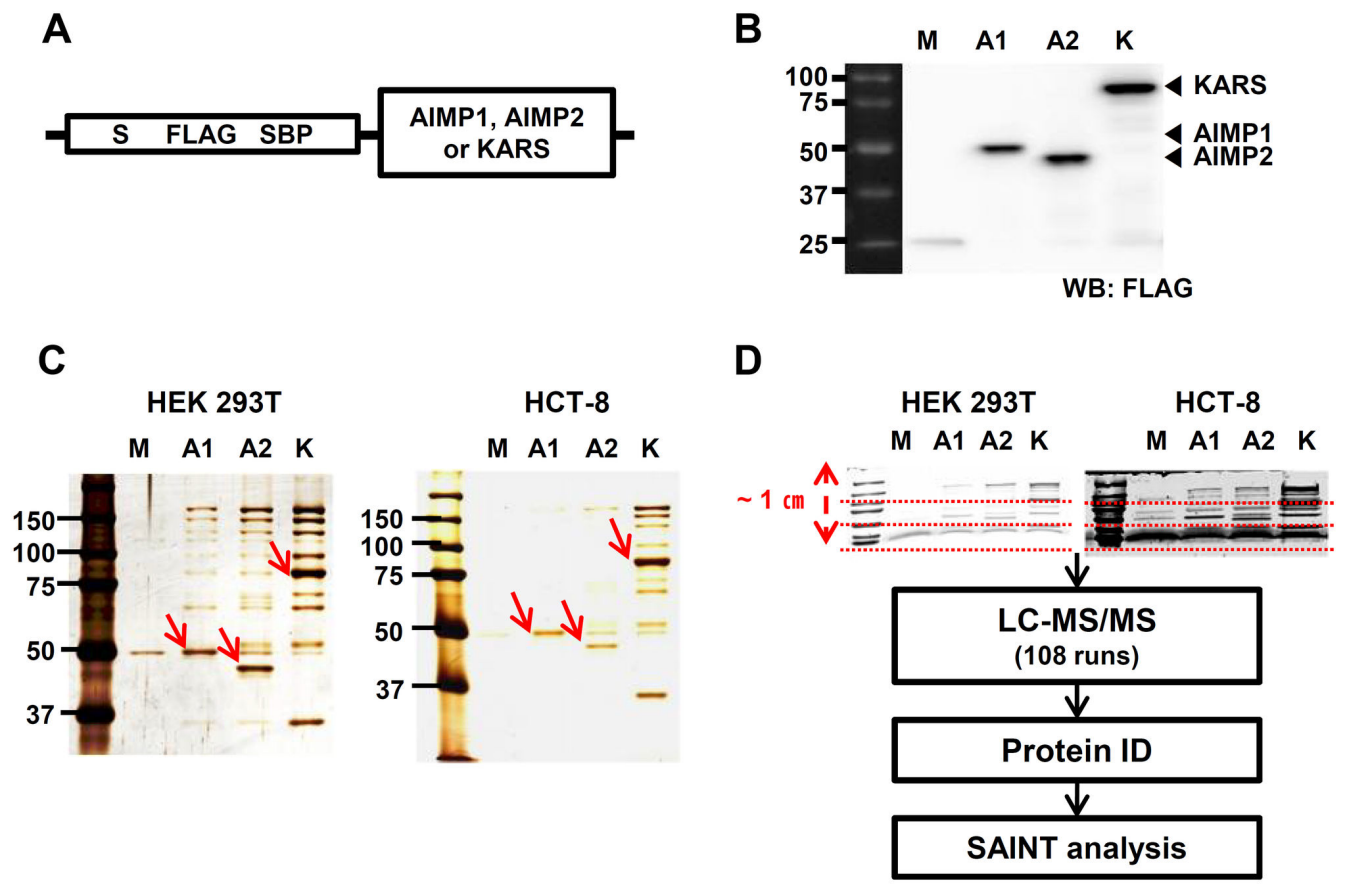
Affinity purification of SBP-tagged AIMP1, AIMP2 and KARS. (**A**) Schematic diagram of AIMP1, AIMP2, and KARS constructs for affinity purification. S/FLAG/SBP tags were attached to the N-terminus of cloned genes. (**B**) Expression of AIMP1, AIMP2, and KARS tagged with S/FLAG/SBP in HEK 293T cells were confirmed by immunoblotting analysis using anti-FLAG antibody. Closed arrowheads (◀) indicate AIMP1, AIMP2 and KARS. (**C**) Streptavidin affinity purification was carried out and 10 % of the eluted samples from HEK 293T and HCT-8 cells were visualized by protein staining. One of three biological replicates is shown and the bait proteins are marked with red arrows. (**D**) 90 % of elution was separated on SDS-PAGE to about 1-cm distance and divided into three fractions each. Then, tryptic peptides were recovered from each gel bands and analyzed by LC-MS/MS. SAINT algorithm was used to calculate the likelihood of true interaction of identified proteins. M; Mock, A1; AIMP1, A2; AIMP2, K; KARS. ‘Mock’ is a vector having the S/FLAG/SBP tag only without target genes.

AIMP1, AIMP2 and KARS were cloned into the vector (pIRES2-EGFP-SBP) containing three types of tags, S, FLAG and SBP (Figure 1A). The tags became fused in-frame at the N-terminus of target genes. Overexpressed S/FLAG/SBP tagged AIMP1, AIMP2 and KARS in HEK 293T cells were immunoblotted by anti-FLAG. The tags increased the molecular weights of the proteins by approximately 15 kDa (Figure 1B). Protein complexes from three biological replicates of AIMP1, AIMP2, KARS and a negative control consisting of the SBP tag alone were purified by streptavidin affinity purification from HEK 293T and HCT-8 cells. One tenth of the eluted proteins were visualized, which confirmed that the experimental approach provided highly purified and reproducible results. Each bait protein such as AIMP1, AIMP2 and KARS showed higher intensity than any other protein band (Figure 1C). The remaining 90% eluted proteins were separated on SDS-PAGE to a short distance and in-gel digests from all gel fractions were analyzed by LC-MS/MS. SAINT algorithm scored probability between bait and preys (Figure 1D). 

A total of 307, 462 and 379 proteins were initially identified from AIMP1, AIMP2 and KARS immunoprecipitates of HEK 293T cells, respectively. Among the proteins, 40, 134 and 101 proteins in each bait appeared to be identified at least twice and to possess a higher than 0.9 average probability from three biological replicates. Similarly, 38, 142 and 84 proteins were recovered from 236, 356 and 305 proteins identified in AIMP1, AIMP2 and KARS immunoprecipitates of HCT-8 cells. All of the identified proteins and their SAINT scores are listed in Table S1 and Table S2. The protein list recovered by SAINT was further used for GO analysis and network analysis.

### Gene Ontology Analysis of AIMP1, AIMP2 and KARS Interactome

**Figure 2:**
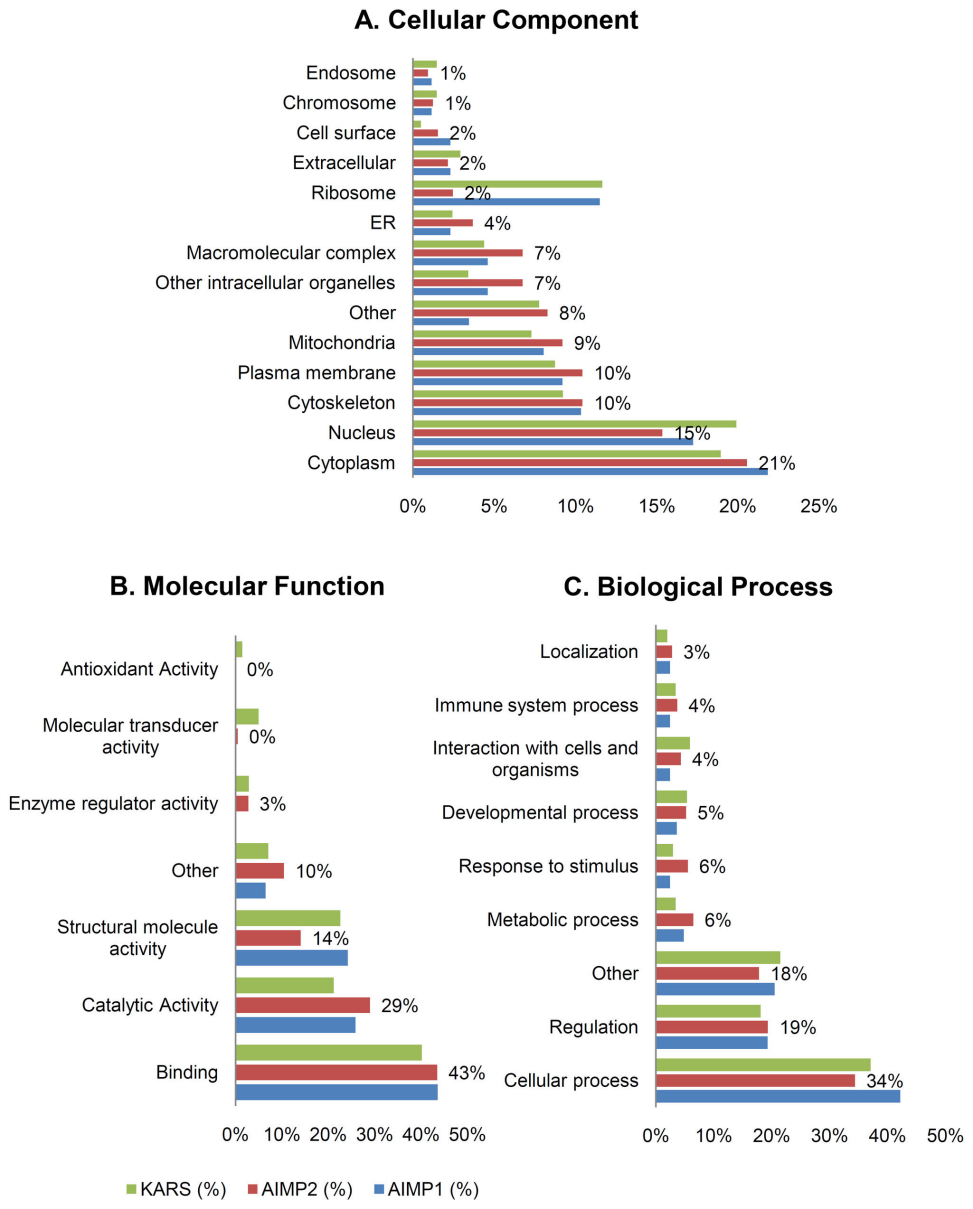
Gene Ontology analysis of AIMP1, AIMP2 and KARS interacting proteins. SAINT analyzed ARS interacting proteins were classified by STRAP (Software Tool for Researching Annotations of Proteins) according to (**A**) cellular component, (**B**) molecular function, (**C**) biological process. The percentage values depicted inside the bar graphs represent AIMP2 results.

To classify the proteins filtered by the SAINT algorithm, we applied GO analysis using STRAP. In the category of cellular components, the proteins were mainly localized to the cytoplasm and nucleus. Ribosome proteins comprised more than 10% of AIMP1 and KARS interactome and this phenomenon occurred in HEK 293T and HCT-8 cells. In contrast, the proportion of ribosome was relatively smaller and various types such as plasma membrane, macromolecular complex and other intracellular organelles existed at a higher rate in AIMP2 interactome (Figure 2A). As expected, more than 40% of the proteins seemed to function in binding. AIMP2 interacting proteins were more likely to have catalytic activity rather than AIMP1 and KARS interacting proteins (Figure 2B). The largest percentage of all three interactomes was associated with cellular process and regulation (Figure 2C). The patterns of these GO analyses were largely similar to the results of HCT-8 cells.

### Network Analysis of AIMP1, AIMP2 and KARS Interactome

**Figure 3:**
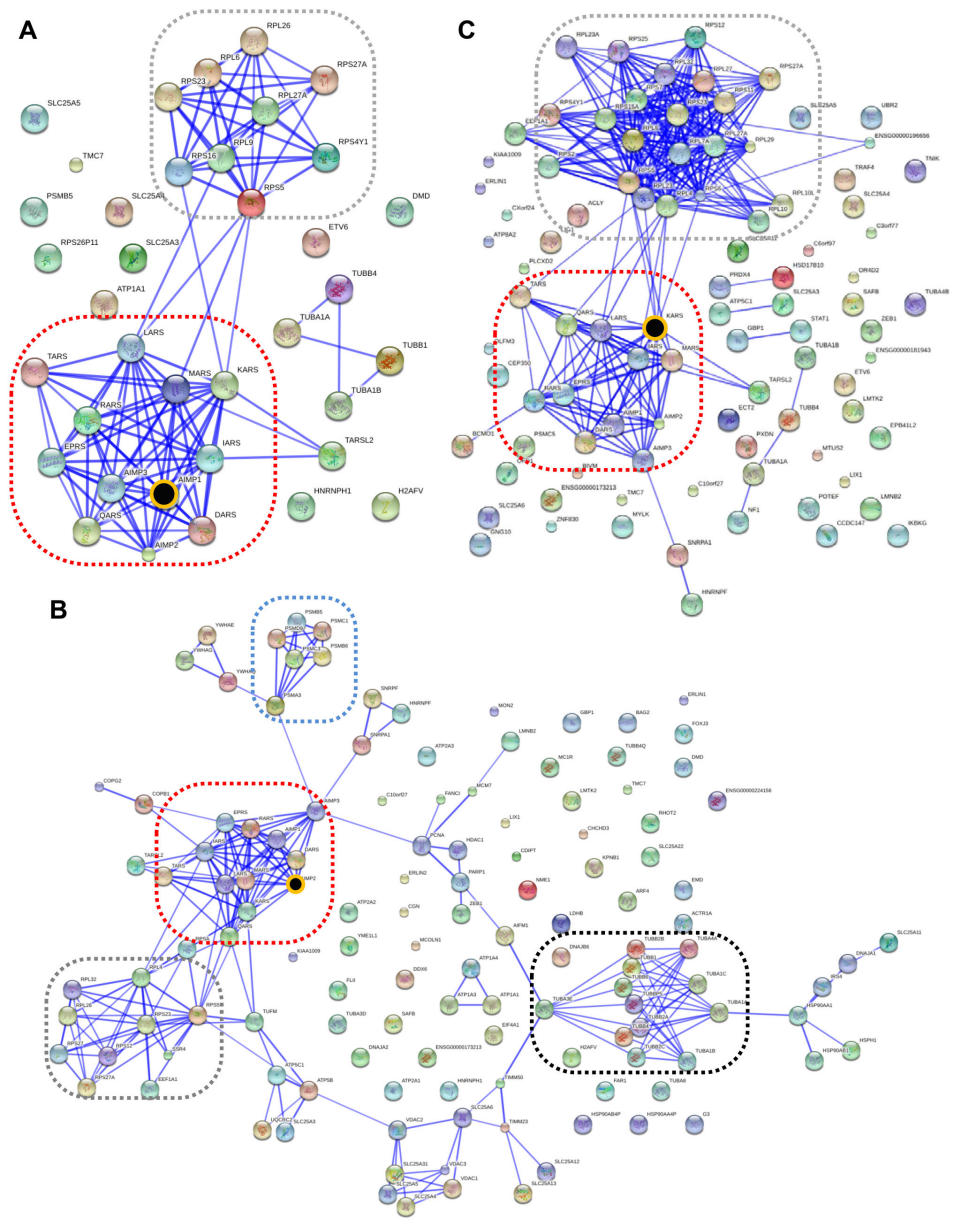
Network analysis of AIMP1, AIMP2 and KARS interactome in HEK 293T cells. Network analyses for AIMP1 (**A**), AIMP2 (**B**) and KARS (**C**) interactome were conducted by STRING 9.05. Red dotted box indicates ARS core-complex comprising AIMPs, D, EP, I, K, L, M, Q and RARS. Ribosomal proteins are grouped in grey dotted box. Blue dotted box shows protein group of proteosome and black dotted box showed tubulin proteins. The baits are indicated with black circles with yellow circumference.

To understand protein associations of ARS complex discovered by our data, the proteins filtered by SAINT were mapped onto protein-protein interaction database by STRING (v9.05). The resultant networks for HEK 293T dataset are shown in Figure 3. ARS proteins previously known as members of the core-complex, such as DARS, EPRS, IARS, KARS, LARS, MARS, QARS and RARS, were constantly covered by all of the three baits. Together with this common characteristic, several distinct features can be found from the networks. First, interacting proteins for AIMP1 were largely grouped in two; one was ARS-core complex proteins and the other ribosomal proteins (Figure 3A). Second, two more groups, heat-shock proteins (HSP) and tubulin proteins were mapped for AIMP2 (Figure 3B). In addition, there were many proteins not belonging to any of these major network groups. AIMP1 and AIMP2 did not appear to be directly linked with various proteins except MSC components. Lastly, a lot more ribosomal proteins were identified for KARS (Figure 3C). Interestingly, TARSL2, which was not known to be a member of MSC, was repeatedly identified and linked with KARS (Figure S1), which guided us to test a possibility that TARSL2 might be a MSC component (see details in later sections).

### Identification of ARS Variants and PTMs

**Figure 4:**
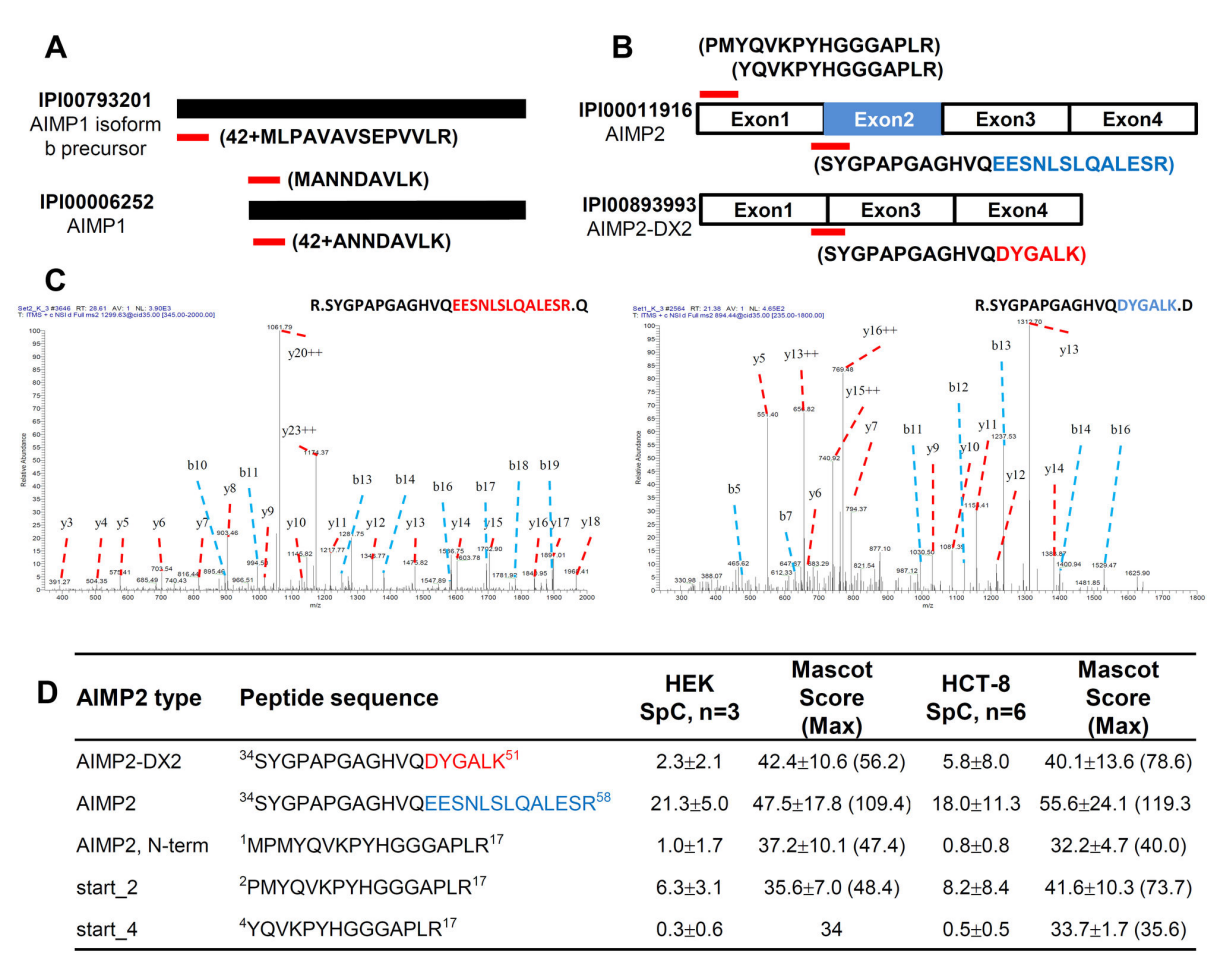
Identification of AIMP1 and AIMP2 variants. (**A**) Canonical AIMP1 and its b precursor isoform. Red bars indicate unique peptide to each isoform. Peptide 42+MLPAVAVSEPVVLR representing isoform b was detected with 3 and 2 spectra in KARS precipitates of HEK 293T and HCT-8 cells. On the other hand, 42+ANNDAVLK representing canonical form had 12 and 20 spectra, respectively. MANNDAVLK which may be an internal peptide of isoform b or N-terminal peptide of canonical form was detected 16 and 15 spectra each. (**B**) Full length AIMP2 and exon-2 deleted AIMP2 (AIMP2-DX2). Two different forms of N-terminal peptide were also identified. Peptides MPMYQVKPYHGGGAPLR and PMYQVKPYHGGGAPLR represent canonical N-terminal part; YQVKPYHGGGAPLR may be a product translated from third residue methionine. (**C**) Peptides representing AIMP2 and AIMP2-DX2 uniquely were identified with significantly different patterns of spectra. SYGPAPGAGHVQEESNLSLQALESR is unique to full-length AIMP2 (left), while SYGPAPGAGHVQDYGALK is unique to AIMP2-DX2 (right). (**D**) AIMP2 and AIMP2-DX2 were identified with 64, 7 and 108, 35 spectra in HEK293T and HCT-8 cells, respectively. Spectral counts and MASCOT scores are indicated as mean±SD and the maximum MASCOT score for each peptide is shown in parenthesis. The experiments were conducted in triplicate on HEK293T cells (n=3) and triplicate of HCT-8 cells were analyzed twice in LC-MS/MS (n=6).

Since the IPI human 3.87 database contains various isoforms of ARS proteins, it was likely to find different isoforms, if any, participating in MSC. Among them, two AIMP proteins were found to exist in isoforms in our data. In addition to the canonical isoforms, AIMP1 isoform 2 and AIMP2-DX2 were identified from KARS precipitates in HEK293T and HCT-8 cells. AIMP1 isoform 2 has 24 additional amino acids at the N-terminus of the canonical sequence (isoform 1) and this was supported by identification of a unique peptide 42+MLPAVAVSEPVVLR corresponding to the isoform 2. We also detected both MANNDAVLK and 42+ANNDAVLK. The latter is attributed to the N-terminal peptide of isoform 1 with initiator methionine removed by methionine aminopeptidase and the second amino acid acetylated by acetyltransferase. The former peptide is considered as either an internal peptide of isoform 2 or N-terminal peptide of isoform 1 with its initiator methionine intact (Figure 4A).

A splicing variant of AIMP2, also known as AIMP2-DX2, was identified with a unique peptide ^34^SYGPAPGAGHVQDYGALK^51^ (Figure 4B and 4C). The peptide unique to AIMP-DX2 was observed reproducibly in the two cell lines with the highest MASCOT ion score of 78.6 and 56.2, respectively (Figure 4D). We also found two different forms of N-terminal peptide of AIMP2. MS/MS spectra for ^2^PMYQVKPYHGGGAPLR^17^ and ^4^YQVKPYHGGGAPLR^17^ peptides counted to 19 and 1 in HEK 293T and 49 and 3 in HCT-8 cells (Figure 4B and 4D). The former peptide is considered as the N-terminal peptide of mature AIMP2, while the latter the N-terminal peptide of another isoform translated from alternative translation initiation site which starts from the third residue methionine.

### Relative Enrichment Analysis of ARS Core-Complex in HEK 293T Cells

**Figure 5:**
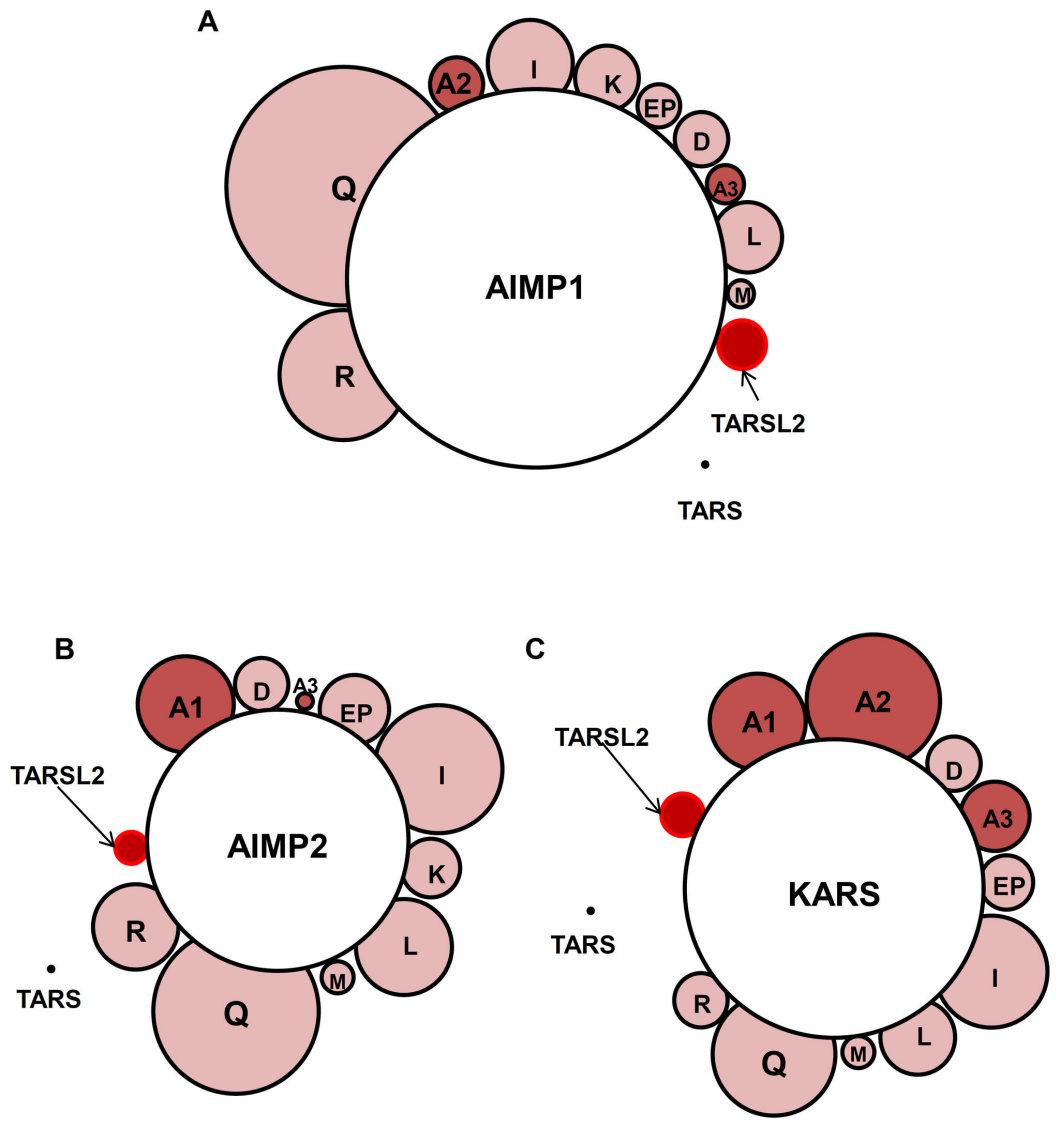
Relative enrichment analysis of ARS core complex proteins in HEK 293T cells. ARS proteins co-purified with by AIMP1 (**A**), AIMP2 (**B**) and KARS (**C**) are represented in the same scale of relative enrichment factors (see the text). Note that TARSL2 appears in all three interactomes with a similar size.

In order to compare the tendency to be co-purified by affinity purification among prey proteins, we observed their NSAF changes between LC-MS/MS data from whole cell lysate of HEK293T and those of AP-MS data. The NSAF of each individual protein in AP-MS data was divided by the NSAF of the protein in whole cell lysate data. Each quotient value was then normalized by dividing it with the average of all quotients for ARS core-complex proteins except bait. We designate this as relative enrichment factor (REF). Though NSAFs are at most semi-quantitative and do not cover the dynamic range linearly, comparing REFs between prey proteins would hint rough estimation about how much prey proteins are involved in MSC. REFs of all complex-forming ARS proteins were close to one, which is self-evident according to the definition of REF, and mostly over 0.3. REFs of bait proteins reached 3 to 5 due to their overexpression for AP-MS. Interestingly, TARSL2 was found in all immunoprecipitates and its REF values were considerably high with 0.57, 0.43 and 0.53 in AIMP1, AIMP2 and KARS immunoprecipitates, respectively. The values were close to those of MARS (0.35, 0.38 and 0.39) and AIMP3 (0.44, 0.18 and 0.84), both of which are known to be members of MSC. TARSL2 was solely ranked on the top of the prey list in the order of REF except for the known MSC components (Figure 5 and Table 1). There were no other proteins with REF values greater than 0.1 in KARS prey lists. Although tubulins and ribosomal proteins formed interaction clusters in AIMP2 and KARS precipitates, the REF values for those proteins were as small as 0.09. In contrast to AIMP1 and KARS, AIMP2 interacted with many proteins whose REFs were greater than 0.1, such as ATP1A, BAG2, DNAJA, ERLIN2, FANCI, FLII, SLC25A, and SSR4 (Table S3) implying that AIMP2 may have various biological roles as well as a scaffold in MSC formation. 

**Table 1:** Proteins having more than 0.3 of Relative enrichment factor (REF).

IPI	Uniprot	Gene Symbol	Protein Name	REF		
				AIMP1	AIMP2	KARS
IPI00006252	Q12904	AIMP1	Aminoacyl tRNA synthase complex-interacting multifunctional protein 1	4.73	1.19	1.18
IPI00011916	Q13155	AIMP2	Aminoacyl tRNA synthase complex-interacting multifunctional protein 2	0.69	3.28	1.65
IPI00216951	P14868	DARS	Aspartyl-tRNA synthetase	0.65	0.72	0.68
IPI00003588	O43324	EEF1E1 (AIMP3)	Eukaryotic translation elongation factor 1 epsilon-1	0.44	0.18	0.84
IPI00013452	P07814	EPRS	Bifunctional aminoacyl-tRNA synthetase	0.55	0.84	0.69
IPI00644127	P41252	IARS	Isoleucyl-tRNA synthetase	1.06	1.60	1.39
IPI00014238	Q15046	KARS	Lysyl-tRNA synthetase	0.80	0.75	3.75
IPI00103994	Q9P2J5	LARS	Leucyl-tRNA synthetase	0.89	1.22	0.95
IPI00008240	P56192	MARS	Methionyl-tRNA synthetase	0.35	0.38	0.39
IPI00925046	P47897	QARS	Glutaminyl-tRNA synthetase	2.95	2.07	1.51
IPI00004860	P54136	RARS	Arginyl-tRNA synthetase	1.62	1.04	0.73
IPI00328082	A2RTX5	TARSL2	Isoform 1 of Probable threonyl-tRNA synthetase 2	0.57	0.43	0.53

Relative enrichment analysis showed that eleven multisynthetase complex proteins, AIMP1, AIMP2, AIMP3, DARS, EPRS, IARS, KARS, LARS, MARS, QARS and RARS, were largely scored over 0.3 of REF in all AIMP1, AIMP2 and KARS precipitates. TARSL2 was scored 0.57, 0.43 and 0.53 in each bait.

### TARSL2 as a Member of ARS Core Complex

**Figure 6:**
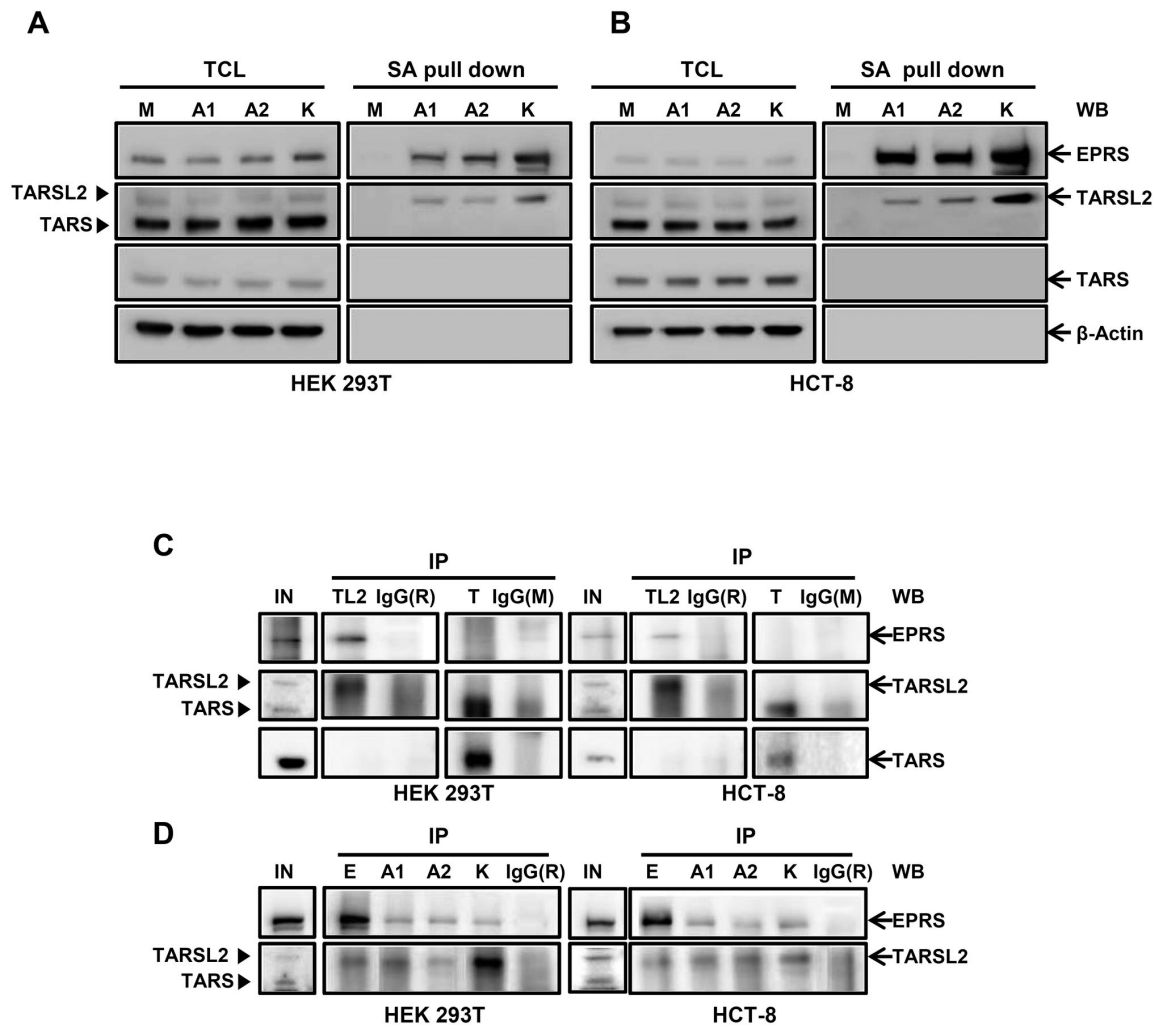
TARSL2 as a member of ARS core complex. (**A**, **B**) TARSL2 and TARS were detected in AIMP1, AIMP2, and KARS immunoprecipitates of HEK 293T (**A**) and HCT-8 cells (**B**). EPRS was used as a positive control. Actin was used for loading control. TCL; total cell lysate, SA pull down; streptavidin pull down. (**C**) Endogenous EPRS was co-immunoprecipitated with TARSL2. Cell lysate (500 μg) was immunoprecipitated with the antibodies against TARSL2, TARS, and IgG and probed for EPRS, TARSL2 and TARS. (**D**) Reciprocal co-immunoprecipitation. Cell lysate (500 μg) was immunoprecipitated with the antibodies against EPRS, AIMP1, AIMP2, KARS and IgG and probed for TARSL2. IgG was used for immunoprecipitation control. M; Mock, E; ERPS, A1; AIMP1, A2; AIMP2, K; KARS, In; Input, TL2; TARSL2, T; TARS, IgG(R); rabbit IgG, IgG(M); mouse IgG. Closed arrowheads (▶) indicate TARSL2 and TARS.

To confirm the interaction between the three ARS core-complex proteins and TARSL2, total cell lysate (TCL) and SA pull down were compared by immunoblotting in HEK 293T and HCT-8 cells (Figure 6A and 6B). The expression level of TARSL2 was similar in TCLs and the protein was also co-purified with the bait protein in AIMP1-, AIMP2- and KARS-transfected cells. Among the two protein bands detected with the anti-TARSL2 antibody in TCL, only the upper band was co-purified with the baits. LC-MS/MS analysis of the protein band (Supplementary Methods in File S1) revealed 46 tryptic peptides specific to TARSL2 (Table S4). On the other hand, the same analysis of tryptic peptides recovered from a gel slice corresponding to the molecular weight of the lower band in TCL sample revealed 24 peptides specific to TARS. The result suggests that anti-TARSL2 antibody we used in this study can detect TARS as well as TARSL2. Anyhow, copurification of the upper band with the baits is a strong indication of TARSL2-bait interaction. It has been reported that TARS is modified by ISGylation in mouse and human cells. This PTM should give a mass increment on TARS of approximately 15 kD [24]. To examine whether the upper band is possibly ISGylated TARS, immunoblotting was performed using anti-ISG15 antibody. There were no detectable bands in SA pull down (Figure S2) indicating that the upper band is not ISGylated TARS, but TARSL2. EPRS, a known MSC component, was also detected in the precipitates (Figure 6A and 6B). In contrast, TARS which is not known as a MSC component was rarely detected in the SA pull down (Figure 6A and 6B).

To confirm endogenous interaction of TARSL2 with other ARS components, coimmunoprecipitation assay was performed. EPRS was detected in the immunoprecipitates of HEK 293T and HCT-8 cells prepared with TARSL2 antibody (Figure 6C). However, the protein was hardly detected in the TARS-immunoprecipitates. The result indicates endogenous interaction of TARSL2 with EPRS. Reversely, TARSL2 was co-immumoprecipitated with endogenous EPRS, AIMP1, AIMP2 and KARS (Figure 6D). Taken together, these data suggest that TARSL2 is a new component of ARS core-complex.

## Comments

Our study aimed to discover the protein-protein interaction of ARS core-complex proteins using a proteomic approach. We performed triplicate affinity purifications and a total of 108 LC-MS/MS runs for the identification of interacting proteins and their post-translational modifications. To reduce the identification of false interactions, two important factors were carefully considered in design of this study. First, affinity-based precipitation using SBP-tag was carried out for purification of interaction partners. SBP-streptavidin binding is known to be strong and very specific [16,25,26]. Second, control immunoprecipitaion using a mock vector containing affinity tag only was used to discriminate bona fide prey proteins from the proteins that would interact with the tag. This filtering was further assisted by the SAINT algorithm that computes confidence scores by comparing the spectral counts of prey for the mock with the spectral counts of prey for baits. Triplicates of affinity purification in HEK 293T and HCT-8 cells showed reproducible results in both SDS-PAGE gels and LC-MS/MS analyses (Figure S3, Table S1 and Table S2). Of twenty ARSs, eight (DARS, EPRS, IARS, KARS, LARS, MARS, QARS and RARS), together with three ARS-Interacting Multifunctional Proteins (AIMPs), are currently known to assemble the multi-synthetase complex. In the affinity-precipitates for each bait (AIMP1, AIMP2 and KARS), all the other components of MSC were confidently found from multiple LC-MS runs. 

AP-MS approach is a powerful technique for interaction proteomics [27,28]. One of the major advantages of AP-MS is that it can be performed under near physiological conditions in the relevant organisms and cell types [29]. Another advantage is that mass spectrometers can detect abundant proteins present in the immunoprecipitate, whether its presence is expected or not. We exploited this feature and found TARSL2 as a new component of ARS-MSC. However, despite these advantages, it has a problem in distinguishing meaningful interactions between bait and prey. For instance, frequent binders such as tubulins, ribosomal proteins and HSPs may undermine specific interactions and the results may be confounded by experimental mistakes and bait specificities. Frequency filter eliminating contaminant proteins and non-specific binding proteins (sticky proteins) may indiscriminately remove frequent binders although they could be a novel interaction depending on the baits [30]. By applying SAINT algorithm, up to 87 % of proteins including a large group of tubulins and ribosomal proteins was unqualified by a threshold of 0.9 and only a few tubulins, ribosomal proteins and HSPs were specifically remained (Figure S4). HSP90AA1, HSP90AB1 and tubulins such as TUBB2A, TUBB2C, TUBB3, TUBB4, TUBB6 and TUBBP5 were identified in AIMP2 precipitates. HSP90 are known to interact strongly with tubulins and serine/threonine protein phosphatase 5 (ref [31].). For AIMP1, unlike AIMP2, only the known components of MSC were qualified after SAINT processing, which implies that AIMP1 has only a defined function as a scaffold of ARS-MSC. 

Recent studies have demonstrated that ARSs and AIMPs interact with various regulatory factors through evolved additional domains [1,5,32]. In addition, a variety of genomic studies on cancers have revealed that many genes are functionally associated with ARSs and AIMPs, which can be defined as cancer-associated genes (CAGs, 123 first neighbors and 1295 second neighbors) [5]. When compared with our datasets, 45 proteins (SAINT prob. ≥ 0.9) were found as CAGs. AIMP2 is known to be involved in cancer progression by interacting p53 on DNA damage or by down-regulating TRAF2 (TNF receptor-associated factor 2) on TNF signal [9,33]. More various prey proteins were identified and mapped in AIMP2 immunoprecipitates than in the other baits (Figure 3). The REF values for such proteins as ERLIN2 (ER membrane lipid raft-associated 2), FANCI (Fanconi anemia complementation group I) and BAG2 (BAG family molecular chaperone regulator 2) were 0.72, 0.72 and 0.3 in only AIMP2 immunoprecipitates unlike those of AIMP1 and KARS (Table S3). Recent studies indicate that ERLIN2 plays roles in supporting cancer cell growth and maintaining transforming phenotypes in breast cancer cells [34,35]. One of Fanconi anemia (FA) proteins, FANCI forms a functional heterodimer by interacting with FANCD2 (Fanconi anemia group D2) and the complex is recruited to the branched DNA structures [36]. On the other hands, FANCI is dissociated from the complex and also functions individually during DNA repair [37]. BCL2-associated athanogene 2 (BAG2) plays a crucial role in cellular senescence in cancer cells by c-Myc-mediated regulation [38]. Thus, AIMP2 seems to be involved in various signaling networks.

KARS is found at various cellular locations also implying various functions [39]. Several proteins identified in KARS interactome (ACLY (ATP citrate lyase) [40], EEF1A1 (elongation factor 1-alpha 1) [41], SAFB (scaffold attachment factor B) [42], SDCBP (syntenin-1) [43], STAT1 (signal transducers and activators of transcription 1) [44], TPM1 (tropomyosin alpha-1 chain) [45] and TRAF4 (TNF receptor-associated factor 4) [46]) were found to be associated with cancer. Except the highly enriched multisynthetase complex proteins, ribosomal proteins were repeatedly identified and dozens of ribosomal proteins were found in KARS immunoprecipitate even though their REF values were smaller than 0.1. They can be considered as sticky proteins which bind with a majority of proteins during translation. But ribosomal proteins were also suggested to function as cell checkpoints and regulators of cell proliferation over protein biosynthesis [47] and it is not unexpected that KARS interacts with translational machinery. Functionally versatile KARS seems to be associated with ribosome biogenesis related to cell proliferation and cancer. 

LC-MS/MS is eligible for identifying both protein modifications such as phosphorylation and isoforms which may be iso-functional or have different functions [48,49]. Translation initiation by ribosome could happen in the downstream AUG codon through leaky scanning [49]. Both AIMP1 and AIMP2 are found to exist in multiple isoforms that have different translation initiation sites (Figure 4A, 4B and 4D). These N-end truncated proteins may have different functions or compensate for the original’s shortage by functioning the same. When the number of spectra representing each N-terminal peptide is simply compared, the expression level of isoforms may be much lesser than the originals. Their functions need to be further investigated. 

In recent studies, AIMP2-DX2, an exon 2-deleted splicing variant, was highly expressed in lung cancer tissue and its suppression consequently reduced tumor growth indicating an important role as a tumor inducing factor [50]. It also competitively inhibited AIMP2 binding to TRAF2, resulting in chemoresistance in ovarian cancer [51]. Such results state that AIMP2-DX2 does not interact with MSC. But our findings imply that it seems to interact with KARS and its expression is detected in HEK293T and HCT-8 cells. We assume that AIMP2-DX2 expresses in various cell types and its expression levels could increase in cancer cells. Relative abundance of full-length AIMP2 may neutralize the effect of AIMP2-DX2 function in cells.

Statistical treatment of MS/MS data by using SAINT and REF demonstrated that threonyl-tRNA synthetase like protein-2 (TARSL2) was likely to be a component of multisynthetase complex. Like threonyl-tRNA synthetase (TARS), TARSL2 presumably catalyzes aminoacylation on cognate tRNA. TARSL2 has approximately 120 conserved amino acids in its N-terminus and has 74% of homology with TARS in whole sequence. We expect that N-terminal region of TARSL2 mediates its binding to ARS core complex. And both of the proteins seem to survive during evolutionary change for efficient protein biosynthesis. As a member of MSC, it may help increase the efficiency of protein biosynthesis or store ARSs to control the non-canonical functions. It is unclear what TARSL2 exactly works for, but our findings overturn the existing knowledge and stimulate further investigation of its potential function as an ARS core-complex protein.

## Supporting Information

Figure S1 **MS/MS spectra, scores and p-values for specific TARSL2 peptides.** Among many spectra matched to TARSL2 peptides, one representative spectrum per each unique peptide is presented. (**A**) PeptideProphet probability, spectrum number, expect value in MASCOT search result, matched ions, peptide sequence, accession number in IPI database and calculated mass for 16 unique peptides were listed. Note that there were three protein entries in IPI database corresponding to TARSL2. The peptides shared with TARS are not shown. (**B**) MS/MS spectra representing TARSL2.
(PDF)

Figure S2 **Detection of ISGylated TARS in HEK 293T and HCT-8 cells.** (**A**, **B**) ISGylated TARS was not detected in AIMP1, AIMP2 and KARS immunoprecipitates of HEK 293T (**A**) and HCT-8 cells (**B**). TCL; total cell lysate, SA pull down; streptavidin pull down.
(PDF)

Figure S3 **Triplicates of affinity purification in HEK293T and HCT-8 cells.** (**A**, **B**) Affinity purification was conducted three times and 10 % of eluted samples were visualized from HEK 293T (**A**) and HCT-8 cells (**B**). Each bait proteins were marked with red arrows. (**C**) 90 % of elution was separated on SDS-PAGE and divided into three fractions. Then, In-gel digests were analyzed by LC-MS/MS. Affinity purifications were conducted reproducibly in three biological replicates. M; Mock, A1; AIMP1, A2; AIMP2, K; KARS.
(PDF)

Figure S4 **SAINT efficiently filtered out non-specific binding proteins.** Grey indicates the number of identified proteins in each bait and interaction partner proteins were distinguishable from the non-specific binding proteins and frequent binders in SAINT analysis. Right y-axis means the percentage of SAINT filtering. The proportion of SAINT filtering was the greatest in AIMP1.
(PDF)

File S1 **Supplementary methods for LC-MS/MS analysis of protein bands.**
(PDF)

Table S1 (A) Identified proteins from AIMP1 precipitated in HEK293T cells and their SAINT scores. (B) Identified proteins from AIMP2 precipitated in HEK293T cells and their SAINT scores. (**C**) Identified proteins from KARS precipitated in HEK293T cells and their SAINT scores.
(XLSX)

Table S2 (A) Identified proteins from AIMP1 precipitated in HCT-8 cells and their SAINT scores. (**B**) Identified proteins from AIMP2 precipitated in HCT-8 cells and their SAINT scores. (**C**) Identified proteins from KARS precipitated in HCT-8 cells and their SAINT scores.
(XLSX)

Table S3 **List of interaction partner proteins having REF values of over 0.1.** ARS core complex proteins were ranked on top.
(XLSX)

Table S4 **List of proteins identified from gel slices corresponding to the molecular masses of TARS and TARSL2.** ‘TCL_upper_band’ labeled tab included the list and information of identified protein in upper band. Peptide information for TARS and TARSL2 was shown in ‘TCL_upper_band_pep’ tab. ‘TCL_lower_band’ labeled tab indicated the list and information of identified protein in lower band. And peptide information for TARS was shown in ‘TCL_lower_band_pep’ tab. ‘SA_pull_down_upper_band’ tab contained the list of identified proteins by SA pull down and TARSL2 specifically identified with 46 unique peptides.
(XLSX)
