# Supplementary material for: Reinvestigation of Aminoacyl-TRNA Synthetase Core Complex by Affinity Purification-Mass Spectrometry Reveals TARSL2 as a Potential Member of the Complex
Source: PLoS One. 2013 Dec 2;8(12):e81734. doi: 10.1371/journal.pone.0081734 (PMC3846882; doi:10.1371/journal.pone.0081734)
Supplement: Figure S1 — MS/MS spectra, scores and p-values for specific TARSL2 peptides. Among many spectra matched to TARSL2 peptides, one representative spectrum per each unique peptide is presented. (A) PeptideProphet probability, spectrum number, expect value in MASCOT search result, matched ions, peptide sequence, accession number in IPI database and calculated mass for 16 unique peptides were listed. Note that there were three protein entries in IPI database corresponding to TARSL2. The peptides shared with TARS are not shown. (B) MS/MS spectra representing TARSL2. (PDF) [file pone.0081734.s001.pdf]

Figure S1. MS/MS spectra, scores and p-values for specific TARSL2 peptides.

Among many spectra matched to TARSL2 peptides, one representative spectrum per each unique peptide is presented. **(A)** PeptideProphet probability, spectrum number, expect value in MASCOT search result, matched ions, peptide sequence, accession number in IPI database and calculated mass for 16 unique peptides are listed. Note that there were three protein entries in IPI database corresponding to TARSL2. Note that the peptides shared with TARS are not shown. **(B)** MS/MS spectra representing TARSL2.

A

prob	spectrum	expect	ions	peptide	protein	calc_mass
0.9986	Set1_K_2.02703.02703.2	0	11/24	R.VADGQTVQGEVWK.T	IPI00328082 +2	1415.7123
0.9985	Set1_K_2.03786.03786.2	0	10/32	K.NSSTYWEGNPEMETLQR.I	IPI00328082 +2	2040.8925
0.9983	Set1_K_1.03856.03856.2	0	18/22	R.MIAILSENYGGK.W	IPI00328082 +2	1294.6669
0.9901	Set2_A1_2.03782.03782.2	0	12/28	R.AVSSTELSALENIC$_{160.03}$K.A	IPI00328082 +1	1620.8107
0.9775	Set2_K_2.05982.05982.2	0.0002	10/24	R.GAFIYNTLTDFIR.E	IPI00328082 +2	1529.7957
0.9789	Set3_K_1.01752.01752.2	0.0001	07/20	K.KGDTSNIITVR.V	IPI00328082 +2	1202.6697
0.9798	Set1_A2_2.02298.02298.2	0	09/22	K.M$_{147.04}$NPGDGAFYGPK.I	IPI00328082 +2	1268.5574
0.9834	Set2_K_2.01050.01050.2	0.0001	09/18	K.VNTATTTVYR.C	IPI00328082 +2	1124.5904
0.9919	Set1_K_2.05070.05070.2	0	11/26	K.QLQNSLMDFGEPWK.M	IPI00328082 +2	1691.8056
0.9668	Set3_A2_1.03067.03067.2	0.0003	09/22	R.NELSGTLSGLTR.V	IPI00328082 +2	1246.6595
0.9879	Set1_A1_1.03515.03515.2	0	13/18	R.IYGISFPDNK.M	IPI00328082 +2	1152.5893
0.9815	Set3_K_1.06120.06120.2	0.0001	15/42	K.TTPYQVAAEISQELAESTVIAK.V	IPI00328082 +2	2348.2189
0.9627	Set1_A1_1.03295.03295.2	0.0004	16/26	R.DFTEVLSPNM$_{147.04}$YNSK.L	IPI00328082 +2	1659.7529
0.9785	Set3_K_1.03255.03255.2	0.0001	08/16	R.FADFGVLHR.N	IPI00328082 +2	1060.5533
0.9597	Set3_K_1.03278.03278.2	0.0005	10/24	R.QVMVIPVGPTC$_{160.03}$EK.Y	IPI00328082 +2	1456.7496
0.6797	Set3_K_1.00265.00265.2	0.0001	08/20	K.MKESEADSEVK.H	IPI00328082 +2	1251.5731

B

NSSTYWEGNPEWETLQR, MH+ 2041.8925, m/z 1021.4499
Set1_K_2.03786.03786.2.dta

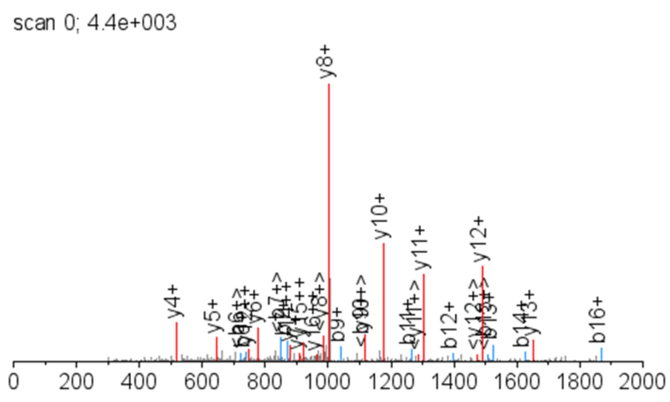

click image to zoom in; click top corners to zoom out

b⁺	#	AA	#	y⁺	y²⁺
115,0502	1	N	17		
202,0822	2	S	16	1927,8491	964,4284
289,1143	3	S	15	1840,8170	920,9124
390,1619	4	T	14	1753,7850	877,3964
553,2253	5	Y	13	1652,7373	826,8726
739,3046	6	W	12	1489,6740	745,3409
868,3472	7	E	11	1303,5947	652,3013
925,3686	8	G	10	1174,5521	587,7800
1039,4116	9	N	9	1117,5306	559,2692
1136,4643	10	P	8	1003,4877	502,2478
1265,5069	11	E	7	906,4349	453,7214
1396,5474	12	M	6	777,3924	389,2001
1525,5900	13	E	5	646,3519	323,6798
1626,6377	14	T	4	517,3093	259,1585
1739,7217	15	L	3	416,2616	208,6347
1867,7803	16	Q	2	303,1775	152,0927
	17	R	1	175,1190	88,0634

VADGQTVQGEVWK, MH+ 1416.7123, m/z 708.8598
Set1_K_2.02703.02703.2.dta

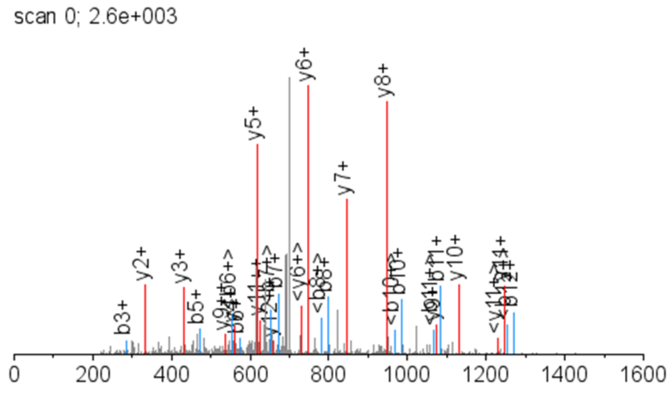

click image to zoom in; click top corners to zoom out

b⁺	#	AA	#	y⁺	y²⁺
100,0757	1	V	13		
171,1128	2	A	12	1317,6434	659,3256
286,1397	3	D	11	1246,6062	623,8070
343,1612	4	G	10	1131,5793	566,2936
471,2198	5	Q	9	1074,5578	537,7828
572,2675	6	T	8	946,4993	473,7535
671,3359	7	V	7	845,4516	423,2297
799,3945	8	Q	6	746,3832	373,6955
856,4159	9	G	5	618,3246	309,6662
985,4585	10	E	4	561,3031	281,1555
1084,5269	11	V	3	432,2605	216,6342
1270,6062	12	W	2	333,1921	167,1000
	13	K	1	147,1128	74,0603

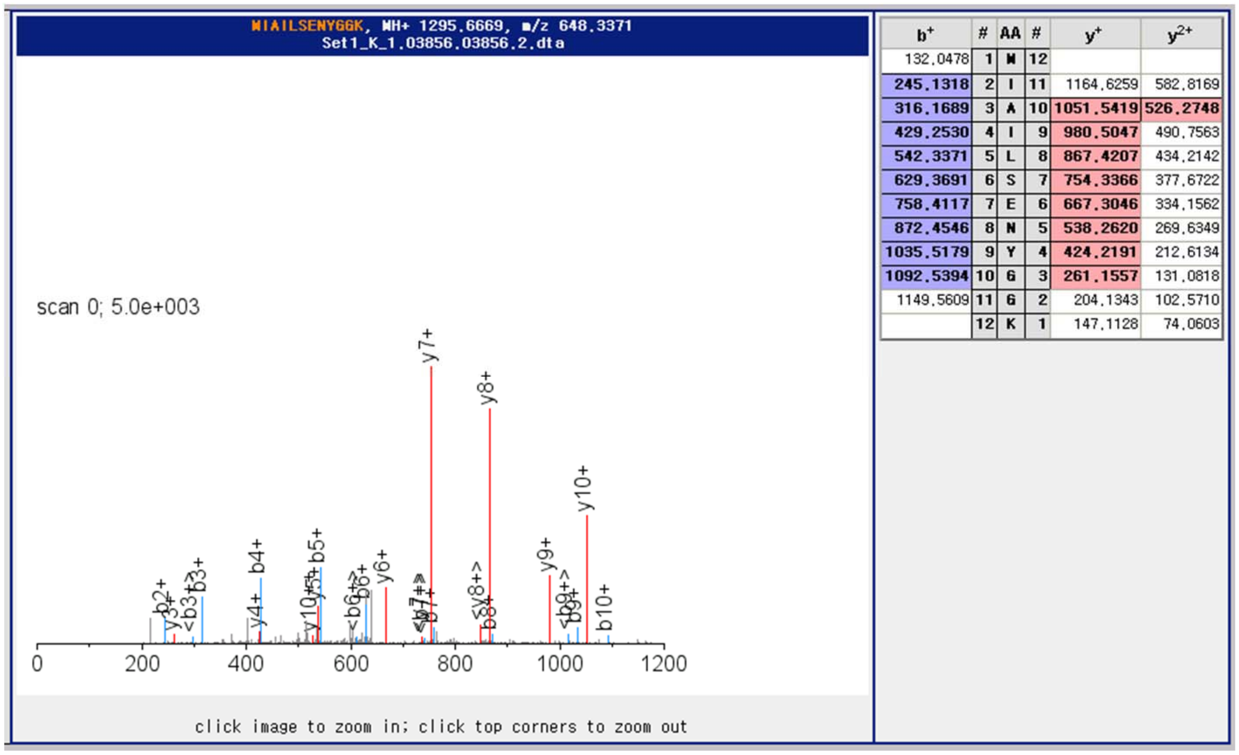

b+	#	AA	#	y+	y2+
132.0478	1	M	12		
245.1318	2	I	11	1164.6259	582.8169
316.1689	3	A	10	1051.5419	526.2748
429.2530	4	I	9	980.5047	490.7563
542.3371	5	L	8	867.4207	434.2142
629.3691	6	S	7	754.3366	377.6722
758.4117	7	E	6	667.3046	334.1562
872.4546	8	N	5	538.2620	269.6349
1035.5179	9	Y	4	424.2191	212.6134
1092.5394	10	G	3	261.1557	131.0818
1149.5609	11	G	2	204.1343	102.5710
	12	K	1	147.1128	74.0603

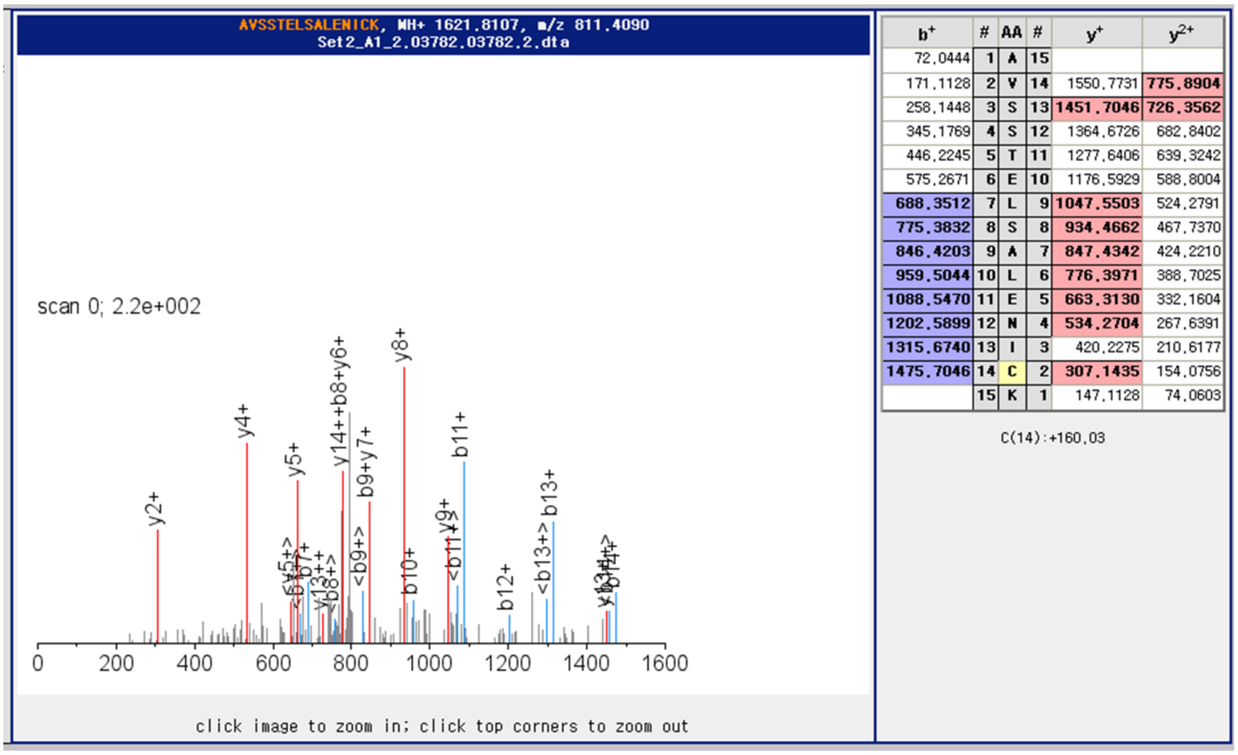

b+	#	AA	#	y+	y2+
72.0444	1	A	15		
171.1128	2	V	14	1550.7731	775.8904
258.1448	3	S	13	1451.7046	726.3562
345.1769	4	S	12	1364.6726	682.8402
446.2245	5	T	11	1277.6406	639.3242
575.2671	6	E	10	1176.5929	588.8004
688.3512	7	L	9	1047.5503	524.2791
775.3832	8	S	8	934.4662	467.7370
846.4203	9	A	7	847.4342	424.2210
959.5044	10	L	6	776.3971	388.7025
1088.5470	11	E	5	663.3130	332.1604
1202.5899	12	N	4	534.2704	267.6391
1315.6740	13	I	3	420.2275	210.6177
1475.7046	14	C	2	307.1435	154.0756
	15	K	1	147.1128	74.0603

C(14):+160.03

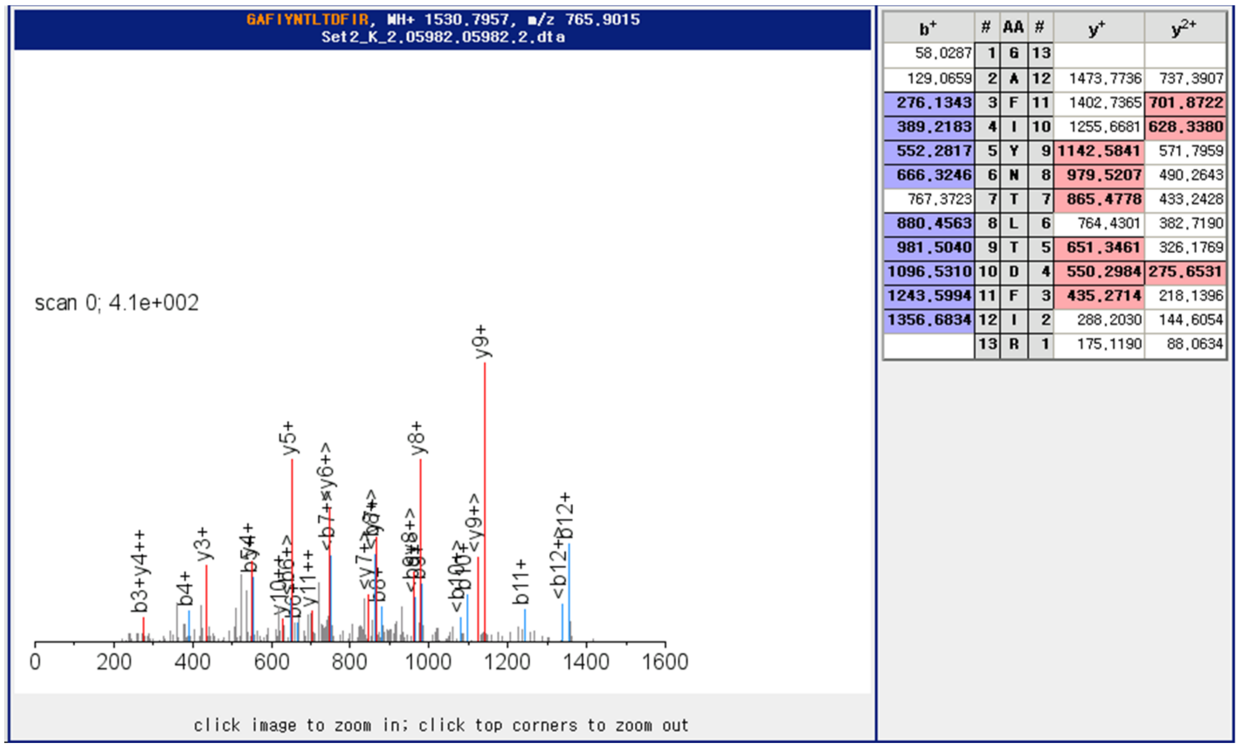

b+	#	AA	#	y+	y2+
58.0287	1	G	13		
129.0659	2	A	12	1473.7736	737.3907
276.1343	3	F	11	1402.7365	701.8722
389.2183	4	I	10	1255.6681	628.3380
552.2817	5	Y	9	1142.5841	571.7959
666.3246	6	N	8	979.5207	490.2643
767.3723	7	T	7	865.4778	433.2428
880.4563	8	L	6	764.4301	382.7190
981.5040	9	T	5	651.3461	326.1769
1096.5310	10	D	4	550.2984	275.6531
1243.5994	11	F	3	435.2714	218.1396
1356.6834	12	I	2	288.2030	144.6054
	13	R	1	175.1190	88.0634

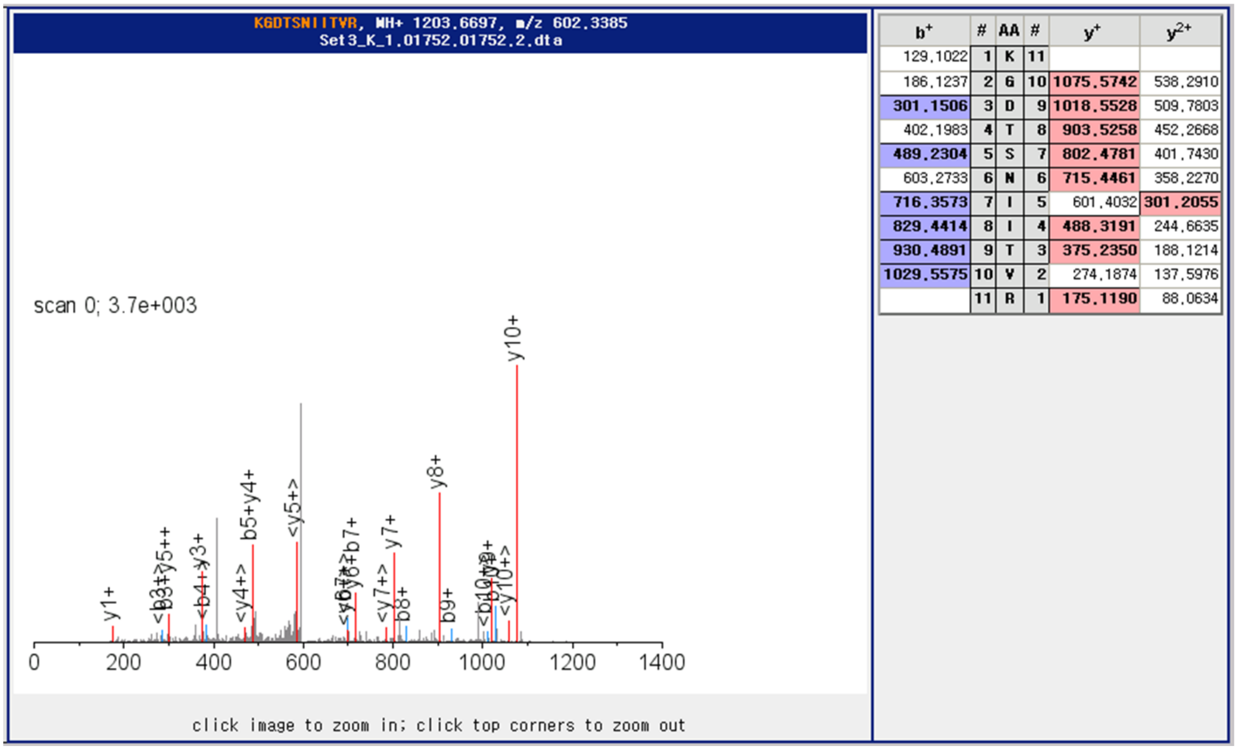

b+	#	AA	#	y+	y2+
129.1022	1	K	11		
186.1237	2	G	10	1075.5742	538.2910
301.1506	3	D	9	1018.5528	509.7803
402.1983	4	T	8	903.5258	452.2668
489.2304	5	S	7	802.4781	401.7430
603.2733	6	N	6	715.4461	358.2270
716.3573	7	I	5	601.4032	301.2055
829.4414	8	I	4	488.3191	244.6635
930.4891	9	T	3	375.2350	188.1214
1029.5575	10	V	2	274.1874	137.5976
	11	R	1	175.1190	88.0634

MNPGDGAFYGPK, MH+ 1269.5574, m/z 635.2823
Set1_A2_2.02298.02298.2.dta

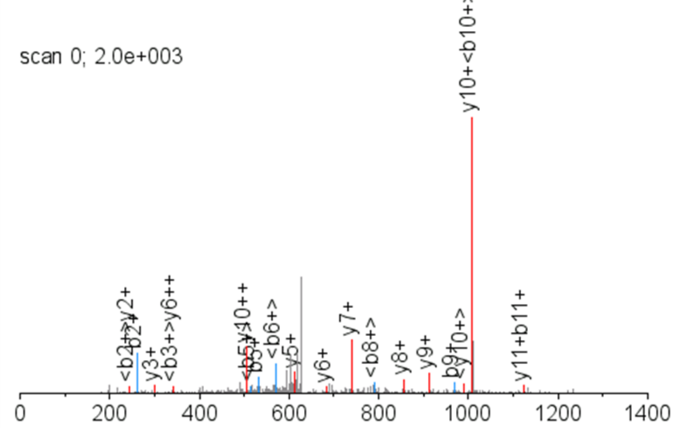

click image to zoom in; click top corners to zoom out

b+	#	AA	#	y+	y2+
148,0427	1	M	12		
262,0856	2	N	11	1122,5215	561,7646
359,1384	3	P	10	1008,4785	504,7432
416,1598	4	G	9	911,4258	456,2168
531,1868	5	D	8	854,4043	427,7061
588,2082	6	G	7	739,3774	370,1926
659,2454	7	A	6	682,3559	341,6819
806,3138	8	F	5	611,3188	306,1633
969,3771	9	Y	4	464,2504	232,6291
1026,3986	10	G	3	301,1870	151,0974
1123,4513	11	P	2	244,1656	122,5867
	12	K	1	147,1128	74,0603

M(1):+147,04

VNTATTTVYR, MH+ 1125.5904, m/z 563.2988
Set2_K_2.01050.01050.2.dta

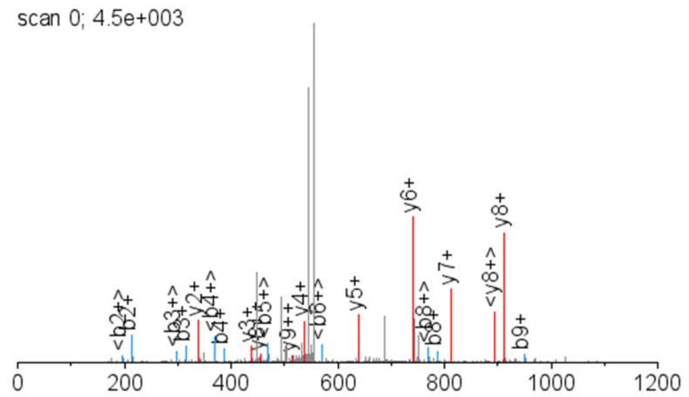

click image to zoom in; click top corners to zoom out

b+	#	AA	#	y+	y2+
100,0757	1	V	10		
214,1186	2	N	9	1026,5215	513,7646
315,1663	3	T	8	912,4785	456,7432
386,2034	4	A	7	811,4308	406,2193
487,2511	5	T	6	740,3937	370,7008
588,2988	6	T	5	639,3461	320,1769
689,3464	7	T	4	538,2984	269,6531
788,4149	8	V	3	437,2507	219,1293
951,4782	9	Y	2	338,1823	169,5951
	10	R	1	175,1190	88,0634

QLQNSLMDFGEPWK, MH+ 1692.8056, m/z 846.9064
Set1_K_2.05070.05070.2.dta

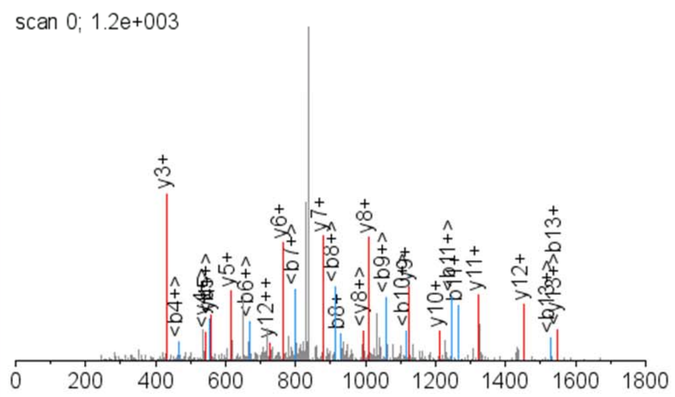

click image to zoom in; click top corners to zoom out

b+	#	AA	#	y+	y2+
129,0659	1	Q	14		
242,1499	2	L	13	1564,7464	782,8771
370,2085	3	Q	12	**1451,6624**	**726,3351**
484,2514	4	N	11	**1323,6038**	662,3058
571,2835	5	S	10	**1209,5609**	605,2843
684,3675	6	L	9	**1122,5288**	561,7683
815,4080	7	M	8	**1009,4448**	505,2263
**930,4349**	8	D	7	**878,4043**	439,7061
1077,5034	9	F	6	**763,3774**	382,1926
1134,5248	10	G	5	**616,3089**	308,6584
**1263,5674**	11	E	4	**559,2875**	280,1476
1360,6202	12	P	3	**430,2449**	215,6264
**1546,6995**	13	W	2	333,1921	167,1000
	14	K	1	147,1128	74,0603

NELSGTLSGLTR, MH+ 1247.6596, m/z 624.3334
Set3_A2_1.03067.03067.2.dta

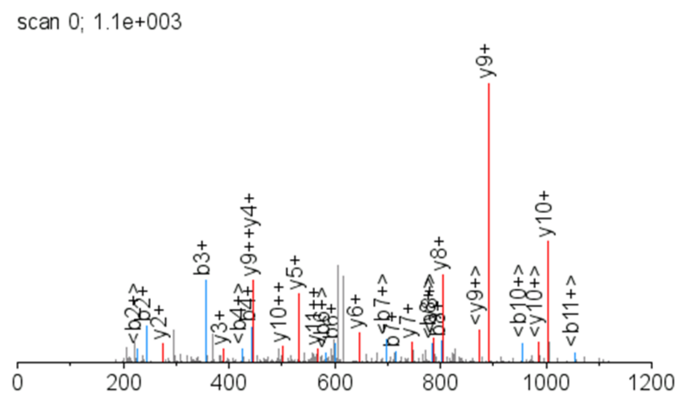

click image to zoom in; click top corners to zoom out

b+	#	AA	#	y+	y2+
115,0502	1	N	12		
**244,0928**	2	E	11	1133,6161	**567,3120**
**357,1769**	3	L	10	**1004,5735**	**502,7907**
**444,2089**	4	S	9	**891,4894**	**446,2486**
501,2304	5	G	8	**804,4574**	402,7326
**602,2780**	6	T	7	**747,4359**	374,2219
**715,3621**	7	L	6	**646,3883**	323,6980
**802,3941**	8	S	5	**533,3042**	267,1560
859,4156	9	G	4	**446,2722**	223,6400
972,4997	10	L	3	**389,2507**	195,1293
1073,5473	11	T	2	**276,1666**	138,5872
	12	R	1	175,1190	88,0634

IYGISFPDNK, MH+ 1153.5893, m/z 577.2983
Set1_A1_1.03515.03515.2.dta

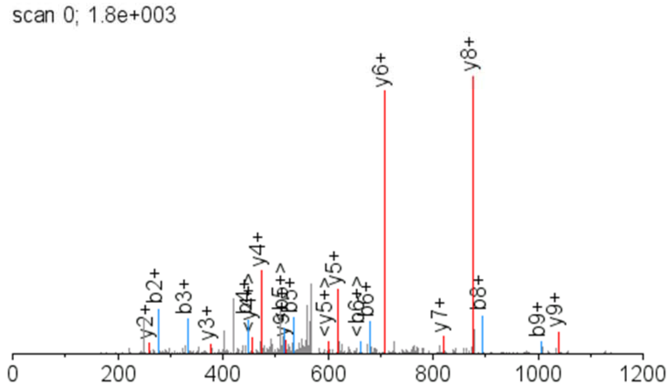

click image to zoom in; click top corners to zoom out

b+	#	AA	#	y+	y2+
114,0913	1	I	10		
277,1547	2	Y	9	1040,5047	520,7563
334,1761	3	G	8	877,4414	439,2246
447,2602	4	I	7	820,4199	410,7139
534,2922	5	S	6	707,3359	354,1719
681,3606	6	F	5	620,3039	310,6558
778,4134	7	P	4	473,2354	237,1216
893,4403	8	D	3	376,1827	188,5952
1007,4833	9	N	2	261,1557	131,0818
	10	K	1	147,1128	74,0603

TTPYQVAAEISQELAESTVIAK, MH+ 2349.2189, m/z 1175.1131
Set3_K_1.06120.06120.2.dta

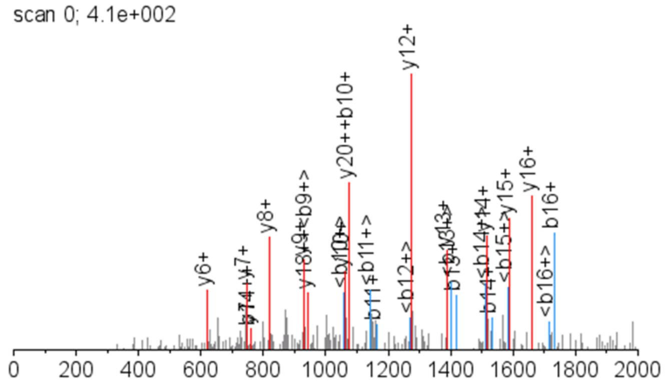

click image to zoom in; click top corners to zoom out

b+	#	AA	#	y+	y2+
102,0550	1	T	22		
203,1026	2	T	21	2248,1707	1124,5893
300,1554	3	P	20	2147,1230	1074,0654
463,2187	4	Y	19	2050,0703	1025,5391
591,2773	5	Q	18	1887,0069	944,0074
690,3457	6	V	17	1758,9484	879,9781
761,3828	7	A	16	1659,8800	830,4439
832,4199	8	A	15	1588,8428	794,9253
961,4625	9	E	14	1517,8057	759,4068
1074,5466	10	I	13	1388,7631	694,8855
1161,5786	11	S	12	1275,6791	638,3434
1289,6372	12	Q	11	1188,6470	594,8274
1418,6798	13	E	10	1060,5885	530,7981
1531,7639	14	L	9	931,5459	466,2768
1602,8010	15	A	8	818,4618	409,7348
1731,8436	16	E	7	747,4247	374,2163
1818,8756	17	S	6	618,3821	309,6950
1919,9233	18	T	5	531,3501	266,1789
2018,9917	19	V	4	430,3024	215,6551
2132,0758	20	I	3	331,2340	166,1209
2203,1129	21	A	2	218,1499	109,5789
	22	K	1	147,1128	74,0603

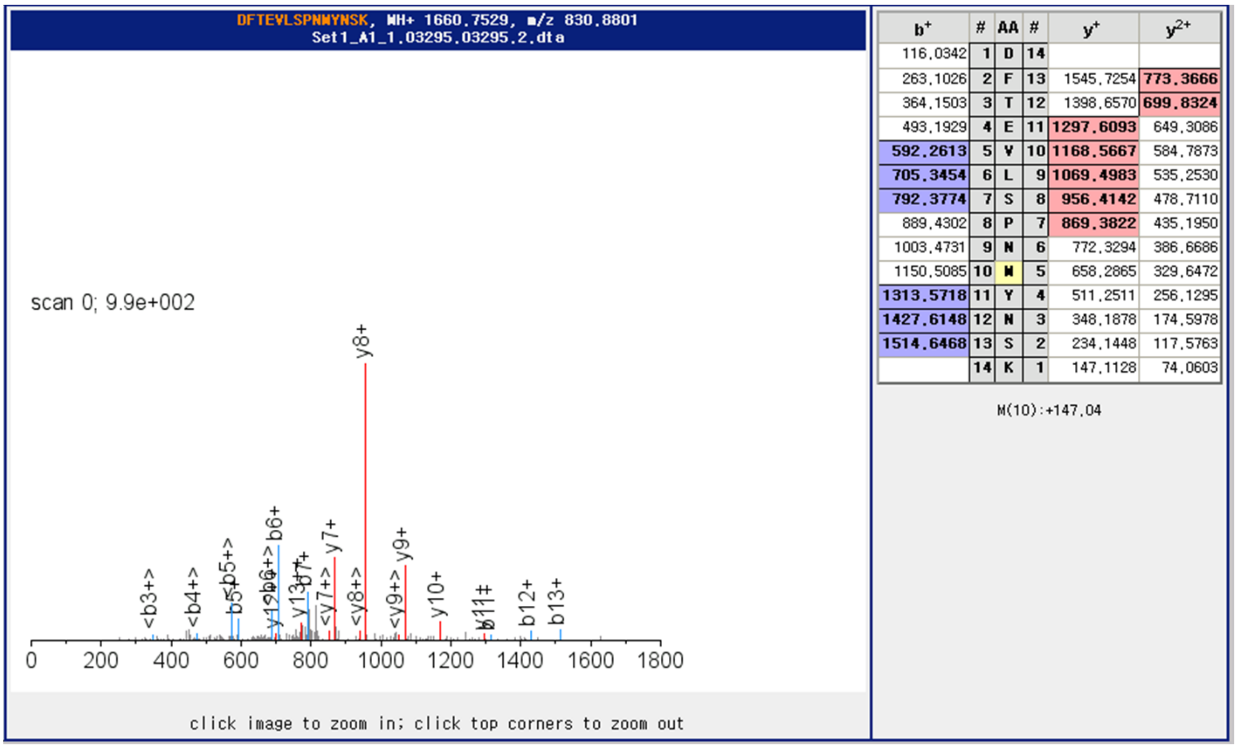

b+	#	AA	#	y+	y2+
116,0342	1	D	14		
263,1026	2	F	13	1545,7254	773,3666
364,1503	3	T	12	1398,6570	699,8324
493,1929	4	E	11	1297,6093	649,3086
592,2613	5	V	10	1168,5667	584,7873
705,3454	6	L	9	1069,4983	535,2530
792,3774	7	S	8	956,4142	478,7110
889,4302	8	P	7	869,3822	435,1950
1003,4731	9	N	6	772,3294	386,6686
1150,5085	10	W	5	658,2865	329,6472
1313,5718	11	Y	4	511,2511	256,1295
1427,6148	12	N	3	348,1878	174,5978
1514,6468	13	S	2	234,1448	117,5763
	14	K	1	147,1128	74,0603

M(10):+147,04

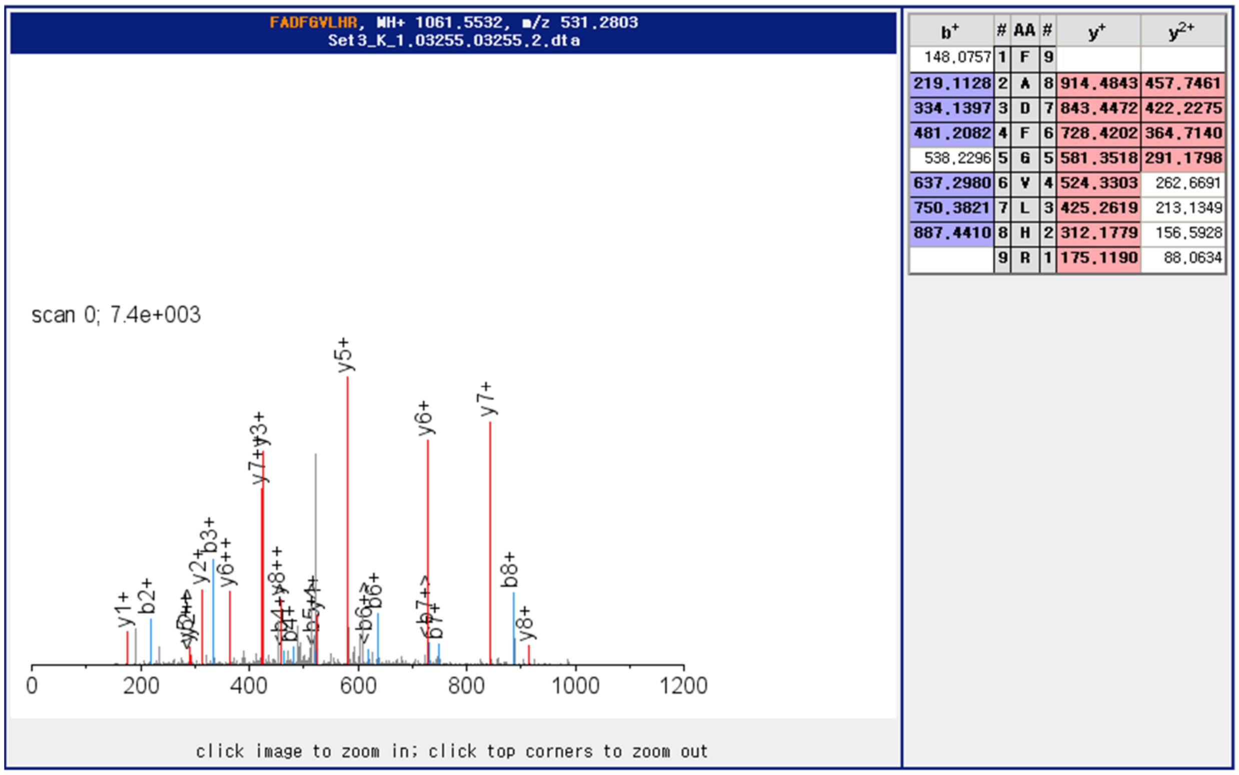

b+	#	AA	#	y+	y2+
148,0757	1	F	9		
219,1128	2	A	8	914,4843	457,7461
334,1397	3	D	7	843,4472	422,2275
481,2082	4	F	6	728,4202	364,7140
538,2296	5	G	5	581,3518	291,1798
637,2980	6	V	4	524,3303	262,6691
750,3821	7	L	3	425,2619	213,1349
887,4410	8	H	2	312,1779	156,5928
	9	R	1	175,1190	88,0634

QVMVIPVGPTCEK, MH+ 1457.7496, m/z 729.3785
Set3_K_1.03278.03278.2.dta

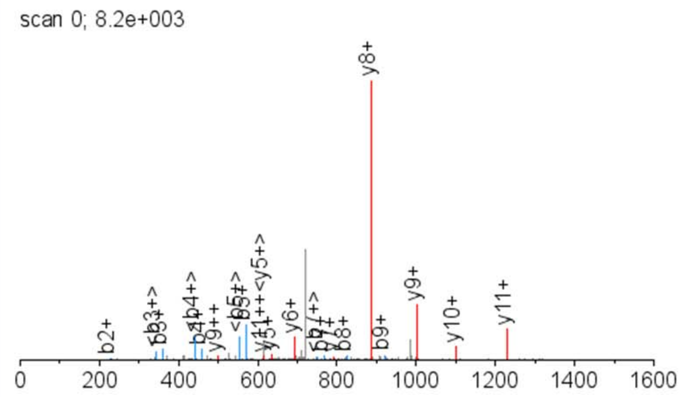

click image to zoom in; click top corners to zoom out

b^+	#	AA	#	y^+	y^{2+}
129,0659	1	Q	13		
228,1343	2	V	12	1329,6905	665,3492
359,1748	3	M	11	1230,6221	615,8150
458,2432	4	V	10	1099,5816	550,2947
571,3272	5	I	9	1000,5132	500,7605
668,3800	6	P	8	887,4291	444,2185
767,4484	7	V	7	790,3764	395,6921
824,4699	8	G	6	691,3080	346,1579
921,5226	9	P	5	634,2865	317,6472
1022,5703	10	T	4	537,2337	269,1208
1182,6010	11	C	3	436,1860	218,5969
1311,6436	12	E	2	276,1554	138,5816
	13	K	1	147,1128	74,0603

C(11):+160,03

MKESEADSEVK, MH+ 1252.5731, m/z 626.7902
Set3_K_1.00265.00265.2.dta

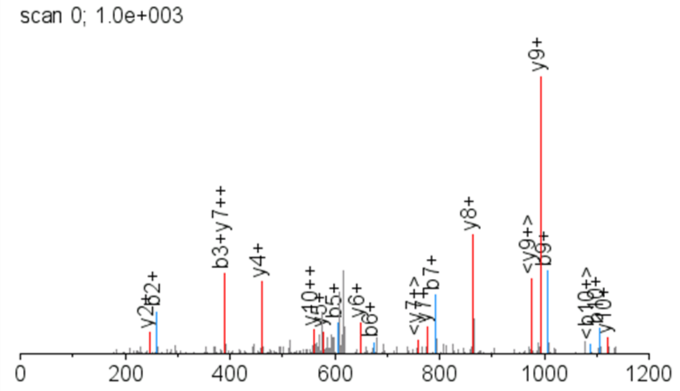

click image to zoom in; click top corners to zoom out

b^+	#	AA	#	y^+	y^{2+}
132,0478	1	M	11		
260,1427	2	K	10	1121,5321	561,2699
389,1853	3	E	9	993,4371	497,2225
476,2173	4	S	8	864,3945	432,7012
605,2599	5	E	7	777,3625	389,1852
676,2971	6	A	6	648,3199	324,6639
791,3240	7	D	5	577,2828	289,1453
878,3560	8	S	4	462,2558	231,6318
1007,3986	9	E	3	375,2238	188,1158
1106,4670	10	V	2	246,1812	123,5945
	11	K	1	147,1128	74,0603
