# Supplementary material for: Reinvestigation of Aminoacyl-TRNA Synthetase Core Complex by Affinity Purification-Mass Spectrometry Reveals TARSL2 as a Potential Member of the Complex
Source: PLoS One. 2013 Dec 2;8(12):e81734. doi: 10.1371/journal.pone.0081734 (PMC3846882; doi:10.1371/journal.pone.0081734)
Supplement: Figure S2 — Detection of ISGylated TARS in HEK 293T and HCT-8 cells. (A, B) ISGylated TARS was not detected in AIMP1, AIMP2 and KARS immunoprecipitates of HEK 293T (A) and HCT-8 cells (B). TCL; total cell lysate, SA pull down; streptavidin pull down. (PDF) [file pone.0081734.s002.pdf]

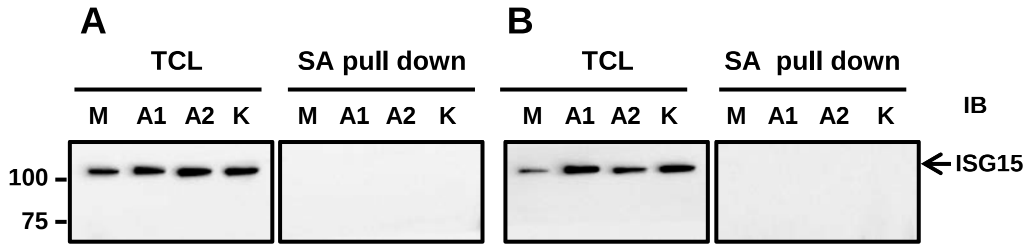

Figure S2. Detection of ISGylated TARS in HEK 293T and HCT-8 cells.

(A, B) ISGylated TARS was not detected in AIMP1, AIMP2 and KARS immunoprecipitates of HEK 293T **(A)** and HCT-8 cells **(B)**. TCL; total cell lysate, SA pull down; streptavidin pull down.
