# Supplementary material for: Reinvestigation of Aminoacyl-TRNA Synthetase Core Complex by Affinity Purification-Mass Spectrometry Reveals TARSL2 as a Potential Member of the Complex
Source: PLoS One. 2013 Dec 2;8(12):e81734. doi: 10.1371/journal.pone.0081734 (PMC3846882; doi:10.1371/journal.pone.0081734)
Supplement: Figure S3 — Triplicates of affinity purification in HEK293T and HCT-8 cells. (A, B) Affinity purification was conducted three times and 10 % of eluted samples were visualized from HEK 293T (A) and HCT-8 cells (B). Each bait proteins were marked with red arrows. (C) 90 % of elution was separated on SDS-PAGE and divided into three fractions. Then, In-gel digests were analyzed by LC-MS/MS. Affinity purifications were conducted reproducibly in three biological replicates. M; Mock, A1; AIMP1, A2; AIMP2, K; KARS. (PDF) [file pone.0081734.s003.pdf]

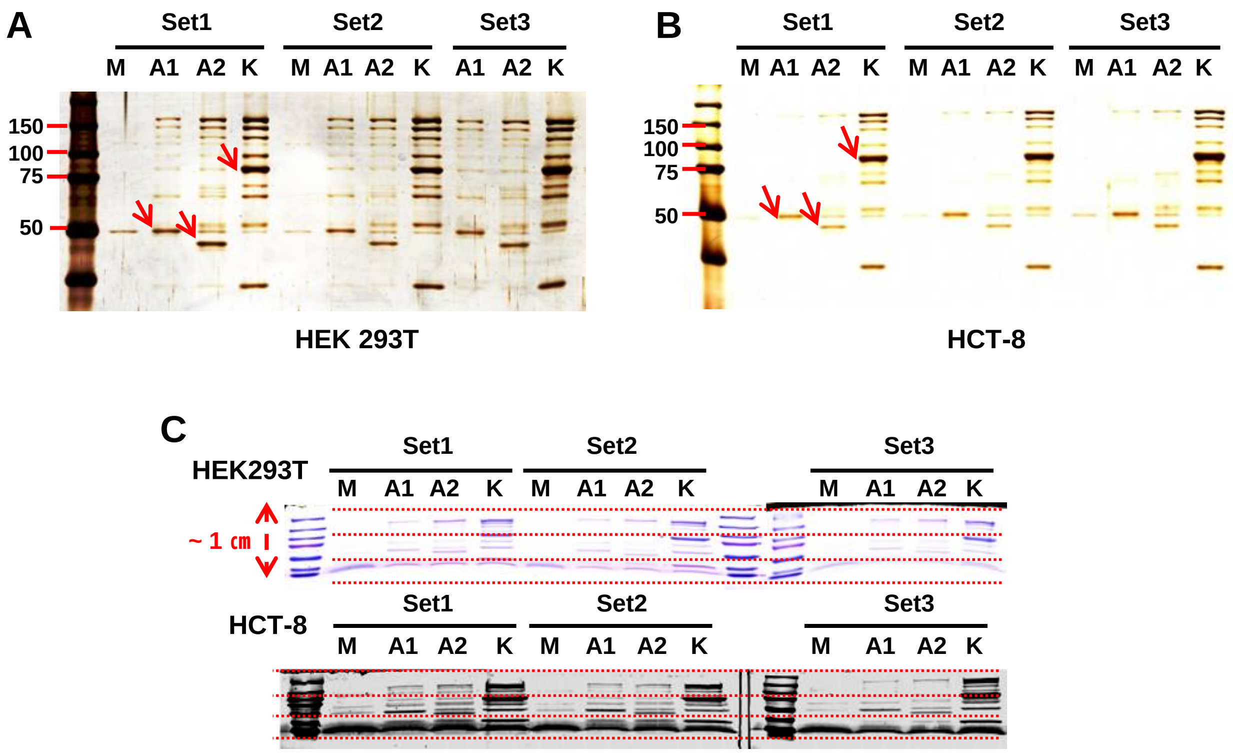

Figure S3. Triplicates of affinity purification in HEK 293T and HCT-8 cells.

(A, B) Affinity purification was conducted three times and 10 % of eluted samples were visualized from HEK 293T **(A)** and HCT-8 cells **(B)**. Each bait proteins were marked with red arrows. **(C)** 90 % of elution was separated on SDS-PAGE and divided into three fractions. Then, In-gel digests were analyzed by LC-MS/MS. Affinity purifications were conducted reproducibly in three biological replicates. M; Mock, A1; AIMP1, A2; AIMP2, K; KARS
