# Supplementary material for: Reinvestigation of Aminoacyl-TRNA Synthetase Core Complex by Affinity Purification-Mass Spectrometry Reveals TARSL2 as a Potential Member of the Complex
Source: PLoS One. 2013 Dec 2;8(12):e81734. doi: 10.1371/journal.pone.0081734 (PMC3846882; doi:10.1371/journal.pone.0081734)
Supplement: Figure S4 — SAINT efficiently filtered out non-specific binding proteins. Grey indicates the number of identified proteins in each bait and interaction partner proteins were distinguishable from the non-specific binding proteins and frequent binders in SAINT analysis. Right y-axis means the percentage of SAINT filtering. The proportion of SAINT filtering was the greatest in AIMP1. (PDF) [file pone.0081734.s004.pdf]

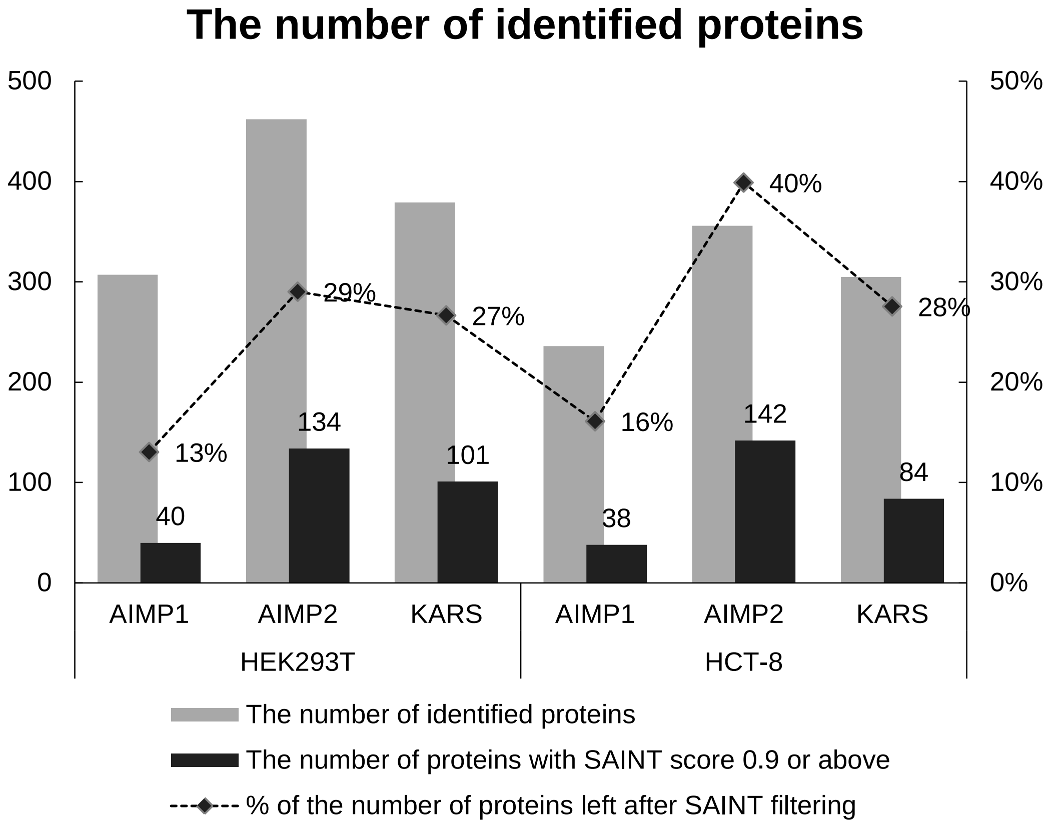


**Figure S4. SAINT efficiently filtered out non-specific binding proteins.**

Grey indicates the number of identified proteins in each bait and interaction partner proteins were distinguishable from the non-specific binding proteins and frequent binders in SAINT analysis. Right y-axis means the percentage of SAINT filtering. The proportion of SAINT filtering was the greatest in AIMP1.
