# Supplementary material for: Reinvestigation of Aminoacyl-TRNA Synthetase Core Complex by Affinity Purification-Mass Spectrometry Reveals TARSL2 as a Potential Member of the Complex
Source: PLoS One. 2013 Dec 2;8(12):e81734. doi: 10.1371/journal.pone.0081734 (PMC3846882; doi:10.1371/journal.pone.0081734)
Supplement: File S1 — Supplementary methods for LC-MS/MS analysis of protein bands. (PDF) [file pone.0081734.s005.pdf]

Supplementary Methods

LC-MS/MS for TARSL2 and TARS

In-gel digestion was performed as described Materials and Methods. The peptide samples were reconstituted in 5 μl of 0.4% acetic acid and 2 μl of each sample was loaded onto a reversed-phase Magic C18aq column (15 cm × 75 μm) on an Eksigent nanoLC-ultra 1D plus system at a flow rate of 300 nL/ min. The column was equilibrated with 95% buffer A (0.1% formic acid in H_2O) + 5% buffer B (0.1% formic acid in acetonitrile) prior to use. Peptides were eluted with a linear gradient from 10% to 40% of buffer B over 40 min. The HPLC system was coupled to a Q Exactive quadrupole mass spectrometer (Thermo Scientific, San Jose, CA, USA). The Q Exactive instrument was operated in the data dependent mode. Full-scan MS spectra (m/z 300–2000) were acquired with a resolution of 75,000, MS/MS spectra of the twelve most intense ions from the MS1 scan with charge state ≥ 2 were acquired with the following options: resolution, 17,500; isolation width, 2 Da; normalized collision energy, 27%; dynamic exclusion duration, 30 s. ion selection threshold, 4.00E+03 counts.

Database Searching and Validation

The acquired MS/MS spectra were searched using the Sequest on Proteome Discoverer (version 1.4, Thermo Fisher Scientific) against the UniprotKB database (May 2013) with 88820 entries. Briefly, precursor mass tolerance was set to ± 15 ppm and MS/MS tolerance was set at 0.05 Da. Cleavage specificity was set to trypsin, allowing for a maximum of two missed cleavages. A variable modification of methionine oxidation (+15.9949 Da) and carbamidomethylated cysteine (+57.0215 Da) was allowed. False discovery rates (FDRs) were set for 1% for each analysis using “Percolator”. From The Sequest search output, Peptide filters which peptide confidence, peptide rank, score versus charge state and search

engine rank were default value of Proteome Discoverer. Protein score threshold was 10 of Sequest.
